# TGF-β1/SMAD3-mediated Non-canonical Hedgehog Signaling Promotes Pancreatic Stellate Cell Activation and Fibrosis in Chronic Pancreatitis

**DOI:** 10.7150/ijbs.108149

**Published:** 2025-10-27

**Authors:** Linrui Peng, Yuchen Hu, Xiaoying Zhang, Chunlu Tan, Chan Yang, Tingting Liu, Pawel E Ferdek, Shufen Yin, Liu Wang, Wei Huang, Yuwei Zhang

**Affiliations:** 1 Department of Endocrinology and Metabolism, Center for Diabetes and Metabolism Research, West China Hospital, Sichuan University, Chengdu, China.; 2 West China Centre of Excellence for Pancreatitis, Institute of Integrated Traditional Chinese and Western Medicine, West China-Liverpool Biomedical Research Centre, West China Hospital, Sichuan University, Chengdu, China.; 3 Division of Pancreatic Surgery, Department of General Surgery, West China Hospital, Sichuan University, Chengdu, China.; 4 Division of Endocrinology and Metabolism, State Key Laboratory of Biotherapy, West China Hospital, Sichuan University and Collaborative Innovation Center of Biotherapy, Chengdu, China.; 5 Department of Cell Biology, Faculty of Biochemistry, Biophysics and Biotechnology, Jagiellonian University, Krakow, Poland.; 6 West China Biobank, West China Hospital, Sichuan University, Chengdu, China.

**Keywords:** chronic pancreatitis, pancreatic stellate cell, Hedgehog signaling, GLI2, SMO, TGF-β1

## Abstract

Excessive Hedgehog (Hh) signaling activity contributes to fibrosis in multiple organs. However, its role in pancreatic stellate cell (PSC) activation and fibrosis development during chronic pancreatitis (CP) remains elusive. We show that GLI2 is one of the top-ranked effectors in the pancreas of CP patients and is highly expressed in activated PSCs. PSC-specific deletion of *Gli2*, but not* Smo*, significantly reduces fibrosis and the severity of the mouse CP, indicating that GLI2 in PSCs can be driven by non-canonical fashion during CP. In culture-activated primary PSCs, early nuclear translocation and increased GLI2 expression are observed promptly following in vitro culture. Whereas GLI2 inhibition reduces PSC activation, SMO inhibition dose not consistently affect changes in GLI2 levels or PSC activation. TGF-β1 promotes GLI2 activation and expression, while these processes and resultant PSC activation are reversed by TGF-β1/SMAD3 inhibition. Altogether, these findings demonstrate the activation of the non-canonical Hh pathway in PSCs during CP and highlight that GLI2 represents a promising therapeutic target for CP.

## Introduction

Chronic pancreatitis (CP) is a progressive inflammatory disease characterized by repeated episodes of acute pancreatitis and persistent inflammation, resulting in extensive fibrosis and impaired pancreatic function.^1^, ^2^ Pancreatic fibrosis is the key pathological hallmark of CP and plays a critical role in its pathogenesis, disease progression, and response to therapy.^3^, ^4^ Once fibrosis is established, the restoration of pancreatic function is limited, rendering the condition irreversible.^5^ Monitoring pancreatic fibrosis progression remains challenging and no effective treatments currently exist to reduce or reverse fibrosis.^6^

Activated pancreatic stellate cells (aPSCs) play a central role in pancreatic fibrosis, producing substantial amounts of extracellular matrix (ECM) components following prolonged exposure to various stimuli.^7^, ^8^ aPSCs originate from quiescent PSCs (qPSCs) through a phenotype change, during which the cells transit from a quiescent, lipogenic state to a fibrogenic and proliferative phenotype.^9^, ^10^ So far, significant progress has been made in understanding the injury-initiated mechanisms that drive PSC activation,^11^-^13^ but key master regulators ultimately regulate PSC fate and related mechanism are yet to be identified and elucidated.

Emerging evidence indicates that Hedgehog (Hh) signaling—a key developmental pathway—also drives a variety of fibrotic diseases.^14^-^17^ In the canonical Hh pathway, Hh ligands (Indian, Sonic, or Desert Hh) bind to the Patched (PTCH) receptor, relieving its inhibition of Smoothened (SMO).^18^ Activated SMO then rapidly accumulates in the primary cilium and launches an intracellular cascade that promotes the activation of GLI transcription factors; the resulting GLI activators translocate into nucleus to drive the expression of Hh target genes. Beyond this canonical ligand-SMO-GLI cascade, there are 3 types of non-canonical Hh signaling pathways operating independently of at least one canonical component. These include PTCH-mediated effects that occur independently of SMO and GLI, SMO-mediated signaling independent of GLI, and direct GLI activation driven by alternative signaling pathways such as the transforming growth factor β / Small Worm and Mothers Against Decapentaplegic (TGF-β/SMAD) signaling.^19^, ^20^ Multiple Hh components, including SMO, PTCH1, and GLI1, are elevated in pancreatic samples from CP patients and animal models,^21^-^23^ and Hh ligands overexpression leads to pancreatic fibrosis in transgenic zebrafish.^24^ Similarly, PSCs can be activated by Sonic Hedgehog (SHH) secreted from pancreatic cancer cells, contributing to perineural invasion and abdominal pain in pancreatic ductal adenocarcinoma (PDAC).^25^, ^26^ Canonical Hh inhibition by SMO inhibitors has failed to show clinical benefit for PDAC patients, highlighting a potential role for non-canonical Hh activation in the pancreas.^27^, ^28^ However, the role of Hh signaling in fibrosis and PSC activation in CP remains rarely explored, and a systematic analysis of canonical versus non-canonical Hh signaling in CP carries significant weight for determining potential therapeutic targets for treating pancreatic fibrosis in CP.

In the present study, we identified GLI2 as the key component of Hh signaling that contributes to pancreatic fibrosis and PSC activation during CP. Although both GLI2 and SMO levels were elevated in aPSCs from CP patients and animal models, only the specific deletion of *Gli2* in PSCs alleviated fibrosis, underscoring the role of non-canonical Hh signaling in fibrosis development during CP. We further demonstrated that non-canonical GLI2 expression and activity occur in response to TGF-β1 signaling, mediated by phosphorylation of SMAD3. These findings indicate that GLI2 plays a critical role in PSC activation, with significant implications for fibrosis development in CP.

## Materials and Methods

### Human specimens

Human pancreas specimens were collected from West China Hospital of Sichuan University. The CP patients exhibited typical clinical features of CP, and the diagnosis was confirmed by computed tomography scans and ultrasound (n = 4). Normal pancreatic tissue specimens were obtained from areas adjacent to duodenal neuroendocrine tumors, at least 2 cm away from the tumor margin (n = 4). This study was approved by the institutional Research Ethics Committee of West China Hospital (No. 2024-933), and all participants signed informed consent prior to their inclusion in the study.

### Reprocessing of SnRNA-Seq and mRNA microarray datasets

We uesd the published pancreatic single-cell data from Tosti and colleagues to explore the expression of Hh signaling in normal and fibrotic human pancreas.^29^ We first used the FindIntegrationAnchors and IntegrateData functions in Seurat (v.4.3.0.1) to remove batch effects from the normal human pancreas (hNP) and CP (hCP) samples and visualized the results of tSNE dimensionality reduction clustering, using the original authors' cell subtype annotations. We visualized differences in gene expression across different cell subtypes using the DotPlot function, averaged gene expression for different type of pancreatic cells using AverageExpression, and plotted heatmaps with the pheatmap package. We also used microarray data Barrera and colleagues to create heatmaps of different types of PSCs in R using the pheatmap package.^30^

### Mice

*Pdgfrb-CreERT2* mice were purchased from the Jackson Laboratory (030201, USA). *Smo*^fl/fl^ and* tdTomato* mice were purchased from the Shanghai Model Organisms Center, Inc. (NM-CKO-200221, NM-KI-225042, China). *Gli2*^fl/fl^ mice were kindly provided by Prof. Lifang Jin (Shaoxing University, China). *Gli2*^fl/fl^*:Pdgfrb-CreERT2* (*Gli2*^△PSC^) or* Smo*^fl/fl^*:Pdgfrb-CreERT2* (*Smo*^△PSC^) mice were generated by crossing *Gli2*^fl/fl^ or *Smo*^fl/fl^ mice with *Pdgfrb-CreERT2* mice. *tdTomato:Pdgfrb-CreERT2* mice were generated by crossing *tdTomato* mice with *Pdgfrb-CreERT2* mice. *C57BL/6J* mice were purchased from the the GemPharmatech Inc. (N000013, China). To generate specific deletion of *Gli2* or *Smo* in PSCs, 4-week-old *Gli2*^fl/fl^, *Gli2*^△PSC^, *Smo*^fl/fl^, *Smo*^△PSC^ mice were intraperitoneally injected with tamoxifen at a dose of 100 mg/kg body weight for 5 consecutive days. Male mice, aged 4 to 10 weeks, were used for the experiments, and were housed under specific pathogen-free conditions. All animal experiments were approved by the Animal Care and Use Committee of Sichuan University (No. 20220624002) and conducted in accordance with the National Institutes of Health's Guide for the Care and Use of Laboratory Animals.

### Bulk RNA-seq analysis

Total RNA was extracted from primary PSCs using TRIzol Reagent (Invitrogen, USA) and subsequently digested with DNaseI. RNA purity and integrity were assessed using a NanodropTM OneCspectrophotometer (Thermo Fisher Scientific, USA) and 1.5% agarose gel electrophoresis, respectively. Quantification was performed with a Qubit 3.0 using a QubitTM RNA Broad Range Assay kit (Life Technologies, China). For library preparation, 2 μg of RNA was processed using the KC-DigitalTM Stranded mRNA Library Prep Kit for Illumina® (Wuhan Seqhealth Co., Ltd., China), which employs an 8-base unique molecular identifier to reduce duplication of polymerase chain reaction (PCR). The resulting 200-500 bp library fragments were sequenced on an Illumina Novaseq 6000 using the PE150 model.

Sequencing data were initially refined by Trimmomatic to remove low-quality and adaptor-contaminated reads, with further deduplication via in-house scripts using unique molecular identifier sequences for clustering. Following pairwise alignment within clusters, reads with over 95% sequence identity were regrouped and realigned to derive consensus sequences, eliminating PCR and sequencing biases.

These de-duplicated sequences were analyzed via standard RNA-seq methods, aligning to the mouse reference genome (GRCm38) using STAR and quantifying gene expression with featureCounts. Differential expression analysis was conducted with edgeR, using a p-value and fold-change threshold of 0.05 and 2, respectively. Gene ontology (GO) enrichment analyses were carried out with KEGG Orthology Based Annotation System, and alternative splicing was examined using replicate multivariate analysis of transcript splicing, both with a stringent false discovery rate cutoff of 0.05.

### CP animal model

One week after tamoxifen injection, the 7-week-old mice received 5 hourly intraperitoneal injections of 100 µg/kg cerulein (Tocris, UK) 3 days per week for a total of 4 weeks to induce CP model.^11^ Mice were sacrificed 3 days after the final cerulein injection. Pancreatic tissue samples were weighed, collected, and prepared for histological and quantitative real-time PCR (RT-qPCR) analyses.

### PSCs isolation and treatment

Primary PSCs were isolated from the pancreas of wild-type or genetically modified mice, following previously described methods.^31^ Briefly, pancreatic tissues were harvested and digested in a solution containing 1.3 mg/mL collagenase P (Roche, Switzerland), 0.01 mg/mL deoxyribonuclease (Roche, Switzerland), and 1 mg/mL protease (Sigma, USA) for 15 mins, then filtered through a 100 µm mesh. After centrifugation, PSCs were purified using 28.7% Nycodenz (Nycomed Pharma, USA) and resuspended in Dulbecco's modified Eagle medium (DMEM)/F12 medium (Gibco, USA) supplemented with 10% fetal bovine serum (FBS; Sigma, USA). Primary PSCs were cultured in DMEM/F12 medium containing 10% FBS, 100 U/mL penicillin, 100 µg/mL streptomycin, and 4 mM L-glutamine, with media changes every 2 days.

After 6 hours of in vitro culture, qPSCs were treated with 10 μM Sonidegib (MCE, USA), 5 μM SIS3 (MCE, USA) or 1 ng/mL TGF-β1 (Preprotech, USA) for 12 h. On the 3^rd^ day of in vitro culture, aPSCs were treated with GANT61 (MCE, USA), 10 μM Sonidegib, 5 μM SIS3 or 1 ng/mL TGF-β1 for 2-4 days. To prove the co-localization of GLI2 and p-SMAD3, primary PSCs were stimulated with 10 ng/mL TGF-β1 for 48 h.

### Lentiviral infection

Lentivirus encoding *Gli2* or *Smo* small interfering RNA (siRNA) were added to the day 3 PSC culture at a multiplicity of infection of 30. PSCs were treated with the lentivirus for 16 hours. 3 days after lentiviral infection, PSCs were harvested for RT-qPCR or immunofluorescence analysis.

### Immunocytochemistry stanning and analysis

After cells were cultured in confocal dishes or 8-well chambers, they were fixed, permeabilized, and blocked. The cells were then incubated overnight at 4°C with primary antibodies at the following concentrations: SMO (1:200, Abcam, ab236465), GLI2 (1:250, Proteintech, 18989-1-AP), GLI1 (1:250, Abcam, ab217326), PTCH2 (1:500, Boster, BA3827-2), ARL13B (1:200, Proteintech, 17711-1-AP), α-SMA (1:250, Sigma, A2547), and Vimentin (1:250, Abcam, ab92547). After washing off the primary antibodies, the cells were labeled with secondary antibodies: Alexa Fluor 488 (1:500, Thermo Fisher, A-11034) or Alexa Fluor 594 (1:500, Thermo Fisher, A21203). Slides were then mounted using a DAPI-containing mounting medium (Sigma, USA).

Images of immunofluorescence-stained cells were captured using a white light confocal microscope (Leica, Germany). The fluorescence parameters set for positive groups were used for capturing images of all subsequent groups stained with the same markers. The 'colocalization' feature in Image*J* software was used to analyze the percentage of colocalization across different channels, including the calculation of the proportion of GLI2 localized in the nucleus and the Pearson's correlation coefficient for GLI2 and p-SMAD3 colocalization. The Pearson's correlation coefficient ranges from -1 to 1, with 0 indicating no colocalization and 1 representing complete colocalization.

### RT-qPCR analysis

Total RNA was isolated from cells or tissues using Trizol reagent (Invitrogen) and complementary DNA (cDNA) was subsequently synthesized by reverse transcription using HiScript III RT SuperMix (Vazyme Biotech, China). RT-qPCR was conducted using the ABI QuantStudio Q6 instrument (ThermoFisher, USA). The relative expression of target genes was calculated using the 2^-ΔΔCt^ method, and data are presented as fold-change relative to the control group. The primers used for RT-qPCR are listed in Supporting Table S1.

### Western blot analysis

Western blot was conducted using established protocols.^32^ Proteins from PSCs were lysed in lysis buffer, and equal protein amounts were subjected to sodium dodecyl sulfate-polyacrylamide gel electrophoresis for separation. Proteins were then transferred onto polyvinylidene difluoride membranes. The membranes were blocked, incubated with antibodies, and the protein bands were visualized. Antibodies used included β-actin (1:10000, Abcam, A1978), Tubulin (1:7500, Proteintech, 66031), GLI2 (1:500, Abcam, ab187386), GLI1 (1:500, Abcam, ab217326), SMO (1:800, Abcam, ab236465), SMAD3 (1:1000, Abcam, ab40854), and p-SMAD3 (1:1000, Santa Cruz, sc-517575). Protein bands were quantified using Image J software and normalized against β-actin or tubulin.

### Co-immunoprecipitation

For co-immunoprecipitation (Co-IP) of endogenous GLI2 and p-SMAD3, primary PSCs were seeded on 10 cm plates and cultured to 70% confluency. Cells were then stimulated with 10 ng/mL TGF-β1 for 48 hours, harvested, and lysed in ice-cold IP buffer (Beyotime, China) containing protease and phosphatase inhibitors (Thermo Fisher, USA). Following centrifugation at 12,000 g for 10 mins at 4°C, the supernatant was collected, and protein concentration was quantified. Total protein of 800 μg per IP reaction was incubated with GLI2 antibody (CST, 18773S) or normal Rabbit IgG (CST, 2729S) overnight on a vertical rotator at 4°C. According to the manufacturer's instructions for the Pierce™ Classic Magnetic IP/Co-IP Kit (Thermo Fisher, USA), the mixture of sample protein and antibody was incubated with 30 µL of pre-washed protein A/G magnetic beads for 1 hour on a vertical rotator at room temperature. After four consecutive washes, the mixture was separated from the protein A/G magnetic beads by a low-pH elution buffer, neutralized with a neutralization buffer, and subsequently analyzed via western blot as previously described.

### Histologic, immunohistochemistry, and immunofluorescence staining and analysis of pancreatic sections

For histologic analysis, pancreatic tissues were fixed in 4% (w/v) paraformaldehyde and embedded in paraffin to and then cut into 3-mm sections. Sections were subjected to hematoxylin and eosin staining (H&E) staining or Masson's trichrome staining and immunohistochemistry stanning for collagen type 1 alpha 1 *(*COL1A1, 1:100, CST, 91144). Pancreatic H&E-, Masson-and COL1A1-stained sections were fully scanned by OLYMPUS VS200 Slide Scanner (OLYMPUS, Japan), and graphically stitched using slide scanner. For each pancreatic sample, five 4x magnified images were randomly captured, and the fibrosis area in Masson stanning was calculated for each image. The average fibrosis area from the five images was taken as the fibrosis area for that sample. The Masson Trichrome channel in the Color Deconvolution feature of Image*J* software was used to decompose the Masson-stained images, and the expression area and proportion of the fibrotic components were calculated.

For pathological scoring of each pancreatic sample, five 4x magnified images were randomly captured, and each image was given a pathological score. The average pathological score of the five images was considered as the pathological score for that sample. The pancreatic H&E pathological scoring was based on the degree of acinar structure atrophy, inflammatory infiltration, pseudoductal complex formation, and the extent of fibrosis.^33^ The first three pathological indicators are scored as follows: 0 points for none, 1 point for mild, 2 points for moderate, and 3 points for severe conditions. For fibrosis, the fibrotic area is first calculated based on Masson's trichrome-stained images, with 0 points for no fibrosis, 1 point if the fibrotic area is < 10%, 2 points if < 20%, and 3 points if < 30%.

Pancreatic tissue sections were stained with primary antibodies as follows: SMO (1:200, Abcam, ab236465), GLI2 (1:200, Proteintech, 18989-1-AP), α-SMA (1:250, Sigma, A2547), and Vimentin (1:250, Abcam, ab92547). Subsequently, these antibodies were labeled with Alexa Fluor 488 (1:500, Thermo Fisher, A-11034) or Alexa Fluor 568 (1:500, Thermo Fisher, A-11011). Images of tissue immunofluorescence-stained sections were captured using a white light confocal microscope (Leica, Germany). Initially, images of CP samples were collected using a 40x objective lens and the parameters were adjusted. The laser intensity and gain for each fluorescence channel were adjusted to ensure the fluorescence was not overexposed, with priority given to adjusting the fluorescence gain to avoid quenching. The fluorescence parameters set for the CP group were used for capturing images of all subsequent sections stained with the same markers. During the capturing process, the microscope's 'zoom in' function was used to magnify and capture images of the target areas. The 'colocalization' feature in Image*J* software was used to analyze the percentage of colocalization across different channels.

### Statistical analysis

Data are presented as mean ± standard error of the mean (SEM). For comparisons between two groups, statistical significance was assessed using a two-tailed Student's t-test. For comparisons among multiple groups, ANOVA followed by a Bonferroni post-hoc test was employed. A p-value less than 0.05 was considered significant for all tests. Specific details about each test, including the p-value and the number of samples analyzed, are included in the figure legends or directly on the figures. All analyses were carried out using Prism Software Version 9.5.0 (GraphPad).

## Results

### GLI2 in PSCs is a key component of Hh signaling in CP

**Figure 1:**
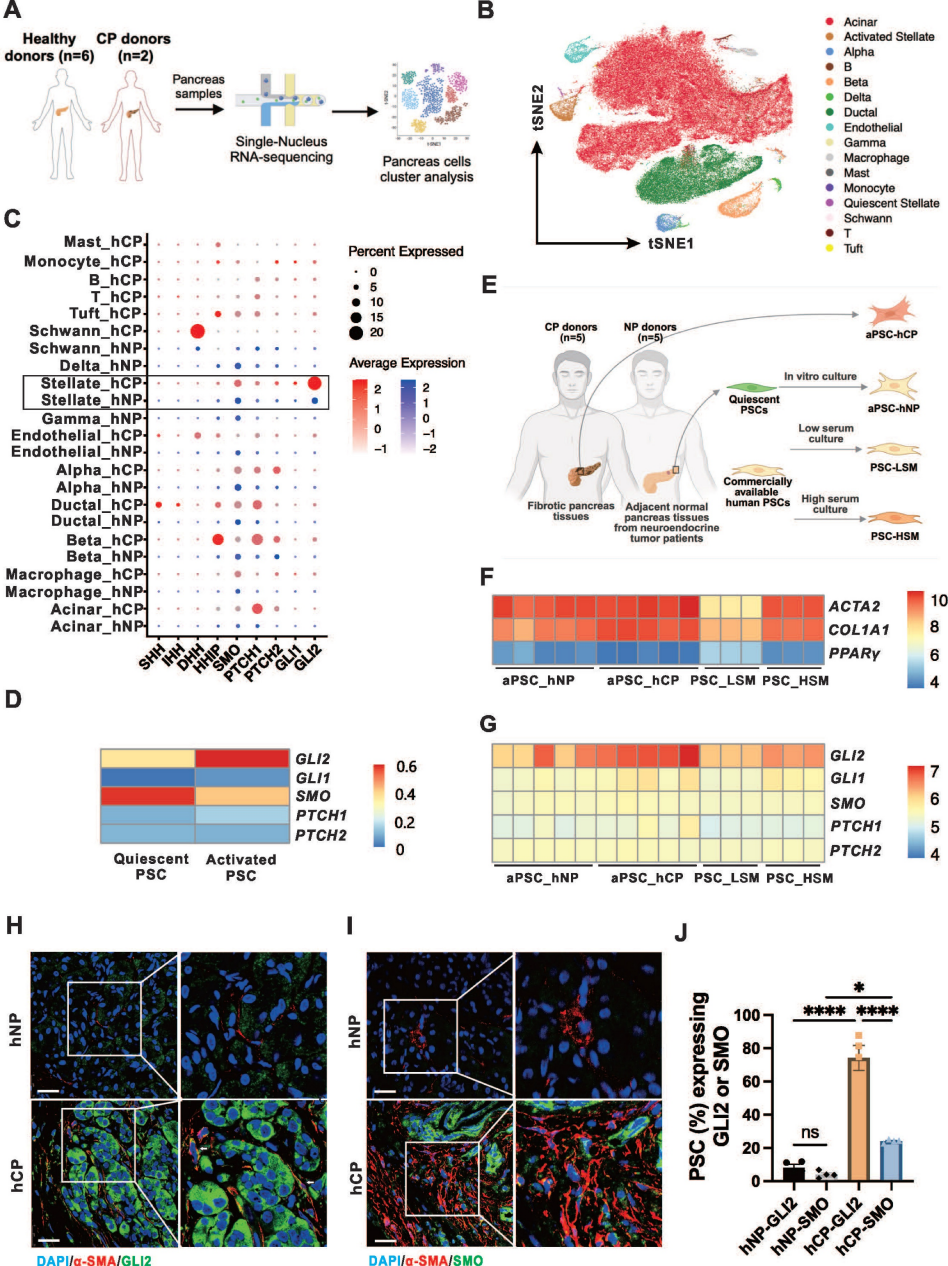
** GLI2 in PSCs is a key component of Hh signaling in CP.** (A) Workflow illustrating for pancreatic snRNA-seq analysis from healthy donors (hNP, n = 6) and CP donors (hCP, n = 2). Data are obtained from Tosti and colleagues.^29^ (B) tSNE plot showing pancreatic cells from hNP and hCP donors, color-coded by cell clusters. (C) Dot plots visualizing the expression levels (color intensity) and frequencies (dot size) of Hh pathway genes across different pancreatic cell clusters from hNP and hCP samples. (D) Heatmap showing the expression patterns of Hh pathway genes in qPSCs and aPSCs. (E) Schematic diagram illustrating the sources and culture conditions of different PSC sample types. Data are derived from Barrera and colleagues.^30^ (F) Heatmap showing expression levels of *ACTA2*, *COL1A1*, and *PPARγ*, indicating varying degrees of activation among the four PSC subtypes. (G) Heatmap displaying the expression profiles of Hh pathway genes across the 4 PSC types. (H, I) Representative dual immunofluorescence images of α-SMA (red) and GLI2 (green) staining in pancreatic tissues from hNP (n = 4) and hCP (n = 4) donors. Nuclei are counterstained with DAPI (blue). Scale bar: 25 µm. (J) Quantification of the proportions of GLI2- and SMO-expressing PSCs in hNP and hCP samples. Data are presented as mean ± SEM. Statistical significance: *p < 0.05, ****p < 0.0001. ns, not significant.

Previous studies have shown increased expression of Hh signaling components in pancreatic tissues from patients and animal models of CP.^21^-^23^ However, the cellular source of these Hh components within the pancreas during CP remains unclear. To investigate the expression of the Hh pathway components in different cell types of the pancreas, we analyzed the single-nucleus RNA sequencing (snRNA-seq) data from hNP (n = 6) and hCP (n = 2) samples (**Figure 1A**).^29^ A total of 2,700 cells from hCP samples and 113,000 cells from hNP samples were clustered, respectively (**Figure 1B**). As shown in the dot plot (**Figure 1C**), Hh signaling components were found to be increased in a number of pancreatic cell types in the hCP samples. Notably, the transcription factor *GLI2* was dramatically upregulated at the mRNA level, particularly in PSCs in CP, while remaining low in other cell types. In addition, *SMO* expression was the most pronounced in PSCs within the CP samples, although its levels were not as elevated as those of *GLI2*. We further examined the mRNA levels of Hh pathway components in PSCs at different activation states. Compared to qPSCs, aPSCs showed slight increases in *GLI1*, *PTCH1*, and *HHIP* expression, with a marked increase in *GLI2* and a decrease in *SMO* expression (**Figure 1D**).

We next analyzed the microarray data to examine the mRNA profiles of PSCs from different sources.^30^ These included PSCs from adjacent normal non-fibrotic pancreatic tissue of neuroendocrine tumor patients (aPSC-hNP), fibrotic pancreatic tissues from CP patients (aPSC-hCP), and commercially available human primary normal PSCs cultured in low serum medium (PSC-LSM) or complete medium (PSC-HSM), respectively (**Figure 1E**). The aPSC-hCP cells were shown to be the most activated among the PSC types, as evidenced by the highest expression levels of smooth muscle alpha-actin 2 (*ACTA2*) and the lowest expression of peroxisome proliferator-activated receptor gamma (*PPARγ*); in contrast, PSC-LSM cells exhibited the lowest activation levels among the four types of PSCs (**Figure 1F**). Further analysis revealed that *GLI2* had the highest expression levels among the Hh receptors and transcription factors, and its expression correlated with the degree of PSC activation. Conversely, the expression levels of *GLI1*, *SMO*, *PTCH1*, and* PTCH2* remained consistent across all 4 types of PSCs (**Figure 1G**).

Given the markedly increased expressions of *SMO* and *GLI2* in PSCs during CP and their respective importance in meditating Hh signaling, we further validated the expression of these proteins within PSCs. Pancreatic tissues from human and experimental CP samples exhibited typical morphological changes, manifesting as extensive pancreatic fibrosis and extensive parenchymal atrophy (**Figure S1A-B**). In accordance with the snRNA-seq data, immunofluorescence imaging showed that the proportion of GLI2- and SMO-expressing PSCs in hCP was significantly elevated compared to hNP samples. Notably, the level of GLI2 expression was approximately 3 times higher than that of SMO (**Figure 1H-J**). This specific pattern of GLI2 and SMO expression in PSCs was further replicated in a mouse CP model (**Figure S1C**).

### PSC-specific deletion of* Gli2* alleviates fibrosis and severity of CP

**Figure 2:**
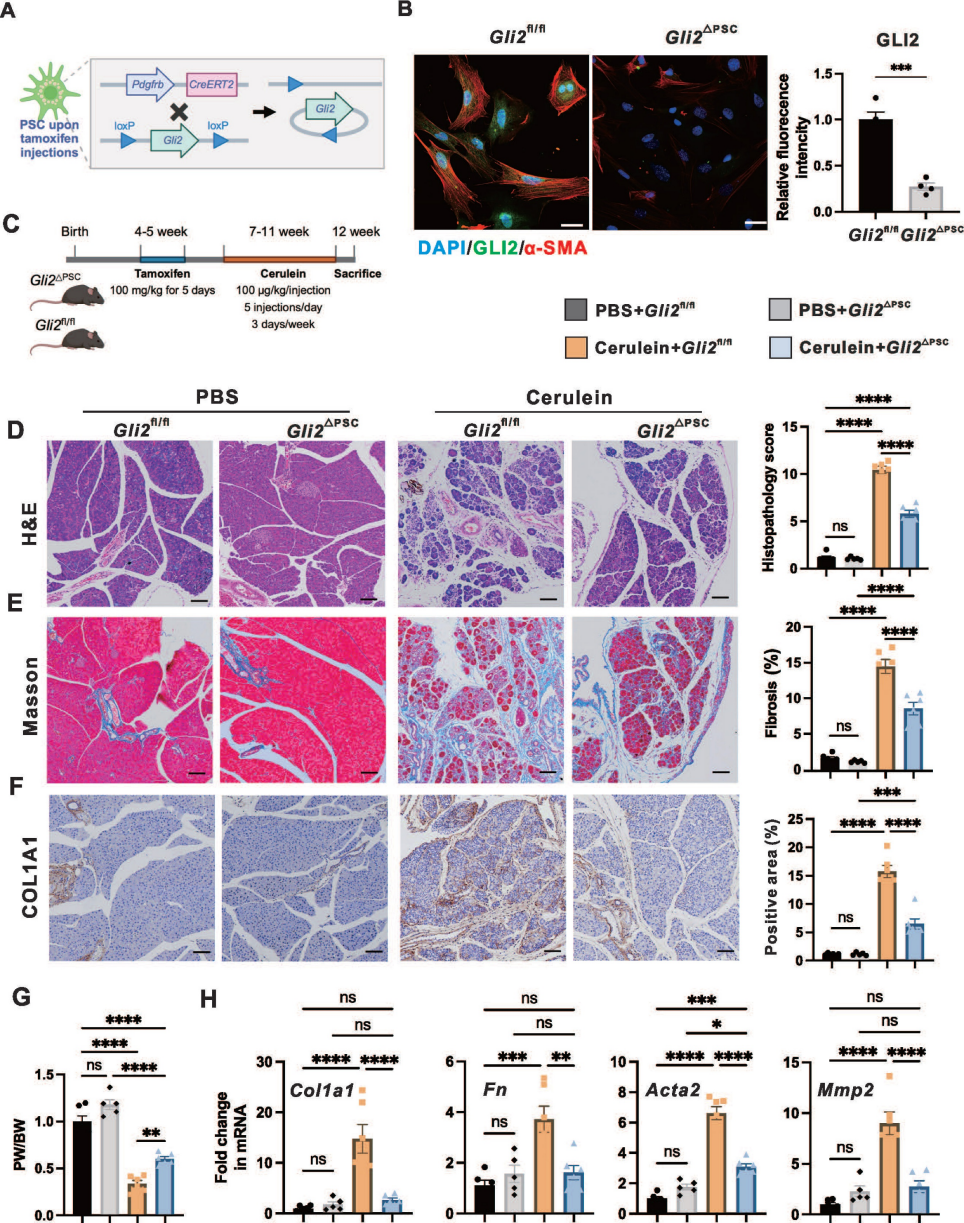
** PSC-specific deletion of* Gli2* alleviates pancreatic fibrosis.** (A) Schematic illustrating the strategy for generating PSC-specific deletion of *Gli2* (*Gli2*^ΔPSC^) in mice. (B) Representative dual immunofluorescence images showing α-SMA (red) and GLI2 (green) in PSCs isolated from *Gli2*^fl/fl^ and *Gli2*^△PSC^ mice. Nuclei were stained with DAPI (blue). Scale bar: 25 µm. GLI2 fluorescence intensity was quantified. (C) Experimental timeline for in vivo induction of CP. (D-F) Representative images of pancreatic sections stained with H&E (D), Masson's trichrome (E), and COL1A1 (F) in* Gli2*^fl/fl^ and *Gli2*^△PSC^ mice treated with PBS or cerulein. Scale bar: 100 µm. Histopathology scores and staining areas were quantified. (G) Pancreatic weight-to-body weight ratio (PW/BW) in* Gli2*^fl/fl^ and *Gli2*^△PSC^ mice treated with PBS or cerulein. (H) Expression levels of fibrotic markers in *Gli2*^fl/fl^ and *Gli2*^△PSC^ mice treated with PBS or cerulein. All data are presented as mean ± SEM. Statistical significance: ^∗^p < 0.05, ^∗∗^p < 0.01,^ ∗∗∗^p < 0.001,^ ∗∗∗∗^p < 0.0001; ns, not significant.

To investigate the role of GLI2 in PSC activation and fibrosis during CP, we generated PSC-specific *Gli2* knock-out mice and assessed the effects of *Gli2* deletion in PSCs on CP progression. Based on previous findings from snRNA-seq data of hNP and hCP samples, platelet-derived growth factor receptor beta (PDGFRB) can serve as a marker for both aPSCs and qPSCs in the pancreas.^29^ To conditionally delete *Gli2* expression in PSCs (*Gli2*^△PSC^), we crossed *Gli2*-floxed mice (*Gli2*^fl/fl^) with *Pdgfrb-CreERT2* mice, where* Cre* expression is controlled by the *Pdgfrb* promoter and induced by tamoxifen administration (**Figure 2A**). We first evaluated CRE recombinase expression in primary PSCs isolated from *tdTomato:Pdgfrb-CreERT2* mice, in which CRE-expressing cells were labeled by tdTomato fluorescence (**Figure S2A**). Intraperitoneal tamoxifen administration to adult *tdTomato:Pdgfrb-CreERT2* mice for 5 consecutive days resulted in reproducible labeling of more than 75% of PSCs (**Figure S2B**). Furthermore, *Gli2* deletion was confirmed by immunofluorescence staining of PSCs derived from *Gli2*^△PSC^ and *Gli2*^fl/fl^ mice, showing a reduction in Gli2 protein expression to 27.2 ± 4.0% in *Gli2*^△PSC^ mice compared to and *Gli2*^fl/fl^ controls (**Figure 2B**). In addition, *Gli2* deletion in PSCs significantly reduced PSC activation, as demonstrated by a marked decrease in α-SMA expression in PSCs from *Gli2*^△PSC^ mice (**Figure 2B**).

Following tamoxifen induced *Gli2* deletion, both *Gli2*^fl/fl^ and *Gli2*^△PSC^ mice underwent repeated cerulein injections to induce CP (**Figure 2C**). Specific ablation of* Gli2* in PSCs significantly alleviated pancreatic morphological changes (atrophy, fibrosis, and inflammation) and their corresponding sum histopathological score (**Figure 2D**). These improvements were further validated by Masson's trichrome staining (**Figure 2E**) and COL1A1 staining (**Figure 2F**), and the pancreas weight ratio (**Figure 2G**). Consistently, *Gli2*^△PSC^ mice exhibited lower expression of fibrotic markers compared to the control groups (**Figure 2H**), indicating a markedly reduced fibrotic phenotype. Collectively, our findings suggest that* Gli2* deficiency in PSCs inhibits PSC activation and substantially decreases the development of fibrosis.

### PSC-specific deletion of *Smo* has minimal impact on fibrosis and severity of CP due to unaffected GLI2 expression

**Figure 3:**
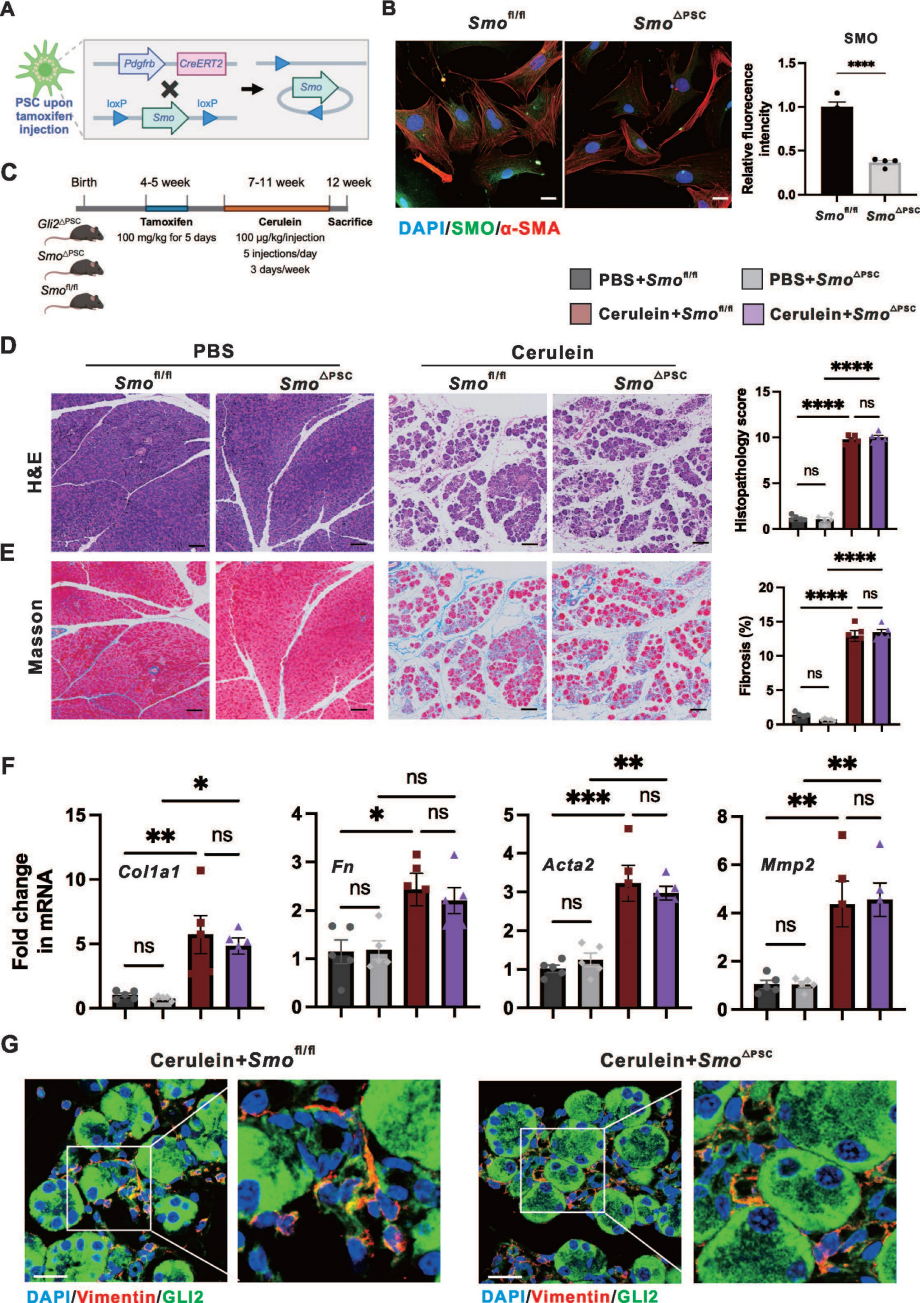
**Specific deletion of *Gli2* but not *Smo* protected against pancreatic fibrosis.** (A) Schematic illustrating the strategy for generating PSC-specific deletion of *Smo* (*Smo*^△PSC^) in mice. (B) Representative dual immunofluorescence images showing α-SMA (red) and SMO (green) in PSCs isolated from *Smo*^fl/fl^ and *Smo*^△PSC^ mice. Nuclei were stained with DAPI (blue). Scale bar: 25 µm. SMO fluorescence intensity was quantified. (C) Experimental timeline for in vivo induction of CP in *Smo*^△PSC^, *Smo*^fl/fl^ and *Gli2*^△PSC^ mice. (D-E) Representative images of pancreatic tissue sections stained with H&E (D), Masson's trichrome (E) in* Smo*^fl/fl^ and *Smo*^△PSC^ mice treated with cerulein. Scale bar: 100 µm. Histopathological scores and fibrotic areas were quantified. (F) Expression levels of fibrotic markers in *Smo*^fl/fl^ and *Smo*^△PSC^ mice treated with cerulein. (G) Representative dual immunofluorescence images showing GLI2 (green) and Vimentin (red) expression in PSCs isolated from *Smo*^fl/fl^ and *Smo*^△PSC^ mice. Scale bar: 25 µm. All data are presented as mean ± SEM. Statistical significance: ^∗^p < 0.05, ^∗∗^p < 0.01,^ ∗∗∗^p < 0.001,^ ∗∗∗∗^p < 0.0001; ns, not significant.

We investigated whether the reduction in fibrosis observed with *Gli2* deletion could be replicated by knocking out the upstream regulator *Smo*, thereby supporting a role for canonical Hh signaling. To generate* Smo* deletion in PSCs (*Smo*^△PSC^), *Smo*-floxed mice (*Smo*^fl/fl^) were crossed with *Pdgfrb-CreERT2* mice (**Figure 3A**). The specific ablation of *Smo* in PSCs resulted in a significant reduction in SMO protein levels to 36.66 ± 2.43% compared to *Smo*^fl/fl^ controls (**Figure 3B**). Further, CP was induced in *Smo*^△PSC^, *Smo*^fl/fl^ and *Gli2*^△PSC^ mice over the same period (**Figure 3C**). Interestingly, *Smo* deletion did not significantly affect fibrosis and severity of CP as demonstrated by pancreatic morphological changes (atrophy, inflammation, and fibrosis) and the overall histopathological score (**Figure 3D**), the extent of fibrotic area (**Figure 3E**), and the expression levels of fibrotic markers (**Figure 3F**). These findings were further supported by the observation that *Gli2*^△PSC^ mice exhibited markedly reduced fibrosis and CP severity compared to *Smo*^△PSC^ mice (**Figure S3A-C**). Additionally, immunofluorescence staining for GLI2 indicated that GLI2 expression in PSCs was not significantly altered between* Smo*^△PSC^ mice and* Smo*^fl/fl^ mice with experimentally induced CP (**Figure 3G**).

We identified and confirmed that in PSCs, GLI2 - but not SMO - is essential for pancreatic fibrosis and disease progression in CP. These findings indicate that GLI2 activation can occur independently of SMO. Therefore, we next investigated the role of non-canonical GLI2 activation in PSC activation.

### GLI2 is essential and active in the early stages of PSC activation

We studied culture-activated primary PSCs from wild-type mice to investigate both early and late events during their phenotypic transition. Freshly isolated PSCs were cultured for 7 days, during which they assumed a typical myofibroblast morphology, accompanied by substantial α-SMA expression by day 7 of in vitro culture (**Figure S4A-B**).

**Figure 4:**
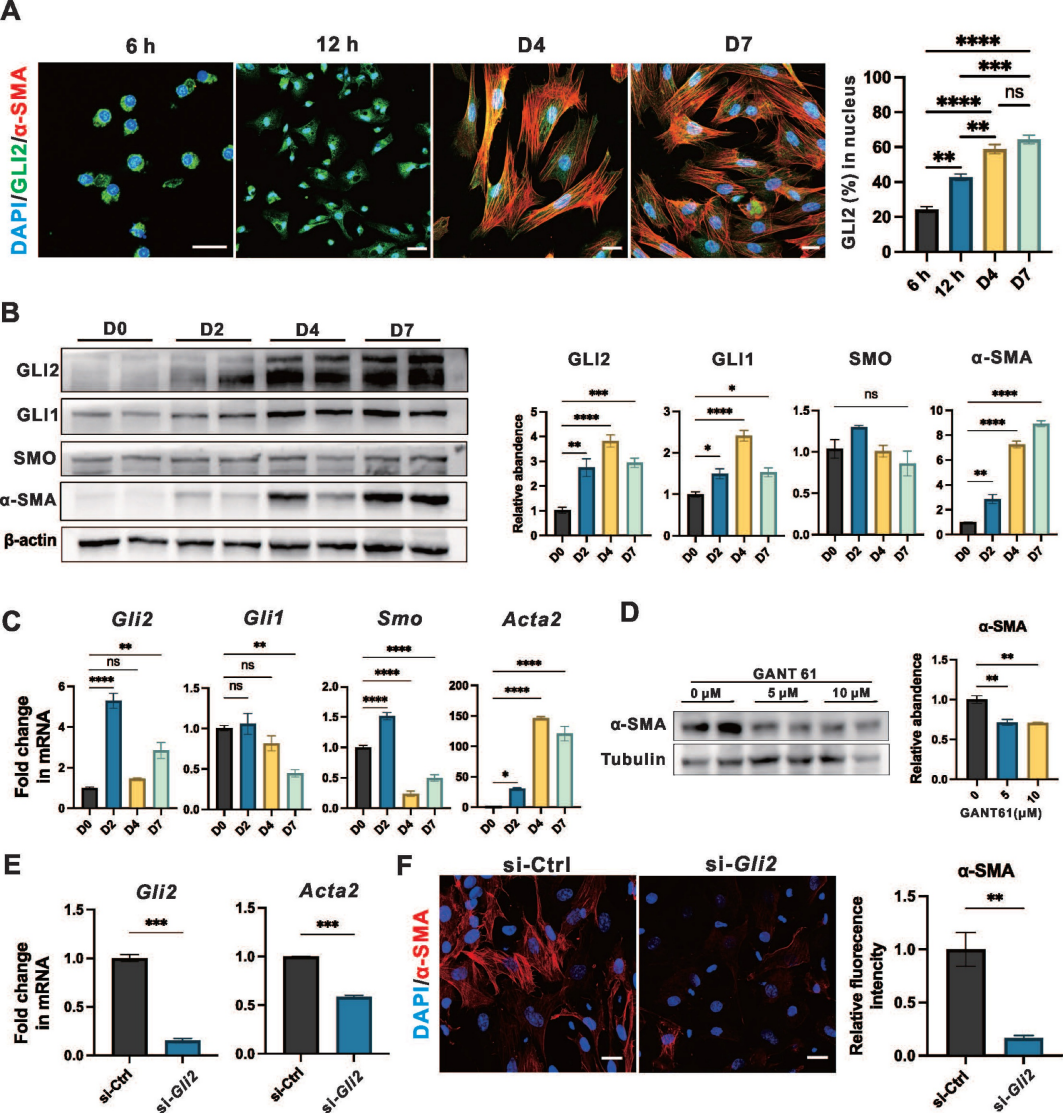
**GLI2 is essential and active during the early stages of PSC activation.** (A) Representative dual immunofluorescence images showing α-SMA (red) and GLI2 (green) in primary PSCs at different culture periods. Nuclei were stained with DAPI (blue). Scale bar: 20 µm. The percentage of GLI2 in the nuclear area was quantified (n = 4). (B) Expression levels of GLI2, GLI1, SMO and α-SMA in primary PSCs at different culture periods. β-actin was used as a normalization control for protein levels (n = 4). (C) mRNA expression levels of *Gli2*,* Gli1, Smo*, and *Acta2* in primary PSCs at different culture periods. *β-actin* was used as a normalization control for mRNA levels (n = 4). (D) Expression levels of α-SMA in primary PSCs treated with GANT61. Tubulin was used as a normalization control for protein levels (n = 3). (E) mRNA expression levels of *Gli2* and *Acta2* in PSCs following *Gli2* silencing.* Gapdh* was used as a normalization control for mRNA levels (n = 3). (F) Representative immunofluorescence images showing α-SMA (red) in PSCs following *Gli2* silencing. Nuclei were stained with DAPI (blue). Scale bar: 20 µm. α-SMA fluorescence intensity was quantified (n = 3). All data are presented as mean ± SEM. Statistical significance: ^∗^p < 0.05, ^∗∗^p < 0.01,^ ∗∗∗^p < 0.001,^ ∗∗∗∗^p < 0.0001; ns, not significant.

GLI2 translocates to the nucleus upon activation, initiating downstream transcriptional programs.^34^ Notably, we observed that GLI2 nuclear translocation occurred at a very early stage of PSC activation - within the first 12 hours of culture (**Figure 4A**). In contrast, the intracellular distribution of SMO and its colocalization with the primary cilium marker ARL13B remained largely unchanged during PSC activation (**Figure S4C-D**). Additionally, the level of GLI2 increased and peaked on day 4, while the level of SMO was constantly unaltered (**Figure 4B**). GLI1, a downstream target of GLI2 and an activator that amplifies the Hh signal,^34^ began to increase on day 2 and peaked on day 4 (**Figure 4B**). Consistent with these observations, the expression levels of *Gli2*, *Smo*, and *Acta2* were all significantly elevated on day 2 (**Figure 4C**). While *Gli2* peaked at day 2 with a greater than 5-fold increase compared to day 0,* Smo* only showed a modest 1.5-fold increase at this time point before declining to less than 0.5-fold thereafter.

To further investigate the role of GLI2 in PSC activation, cells were treated for 4 days with two concentrations of GANT61, a GLI inhibitor that blocks the binding of GLI proteins to their target DNA sequences. Both treatments led to a significant reduction in α-SMA expression (**Figure 4D**), while the expression of GLI2 remains unchanged (**Figure S4E**). Additionally, lentiviral transduction with small interfering RNA (siRNA) targeting* Gli2* (si-*Gli2*) was used to silence GLI2 expression (**Figure 4E**). Treatment with si-*Gli2* significantly reduced α-SMA expression compared to control siRNA (si-Ctrl) treatment (**Figure 4F**).

Collectively, these data suggest that during the early stages of PSC activation, GLI2 undergoes nuclear translocation, accompanied by a dramatical increase in its expression level. Inhibiting GLI2 function results in decreased PSC activation, as indicated by reduced α-SMA expression. In contrast, SMO expression and localization remained unchanged throughout the PSC activation process.

### SMO inhibition is insufficient to affect long-term expression of GLI2 in PSCs

**Figure 5:**
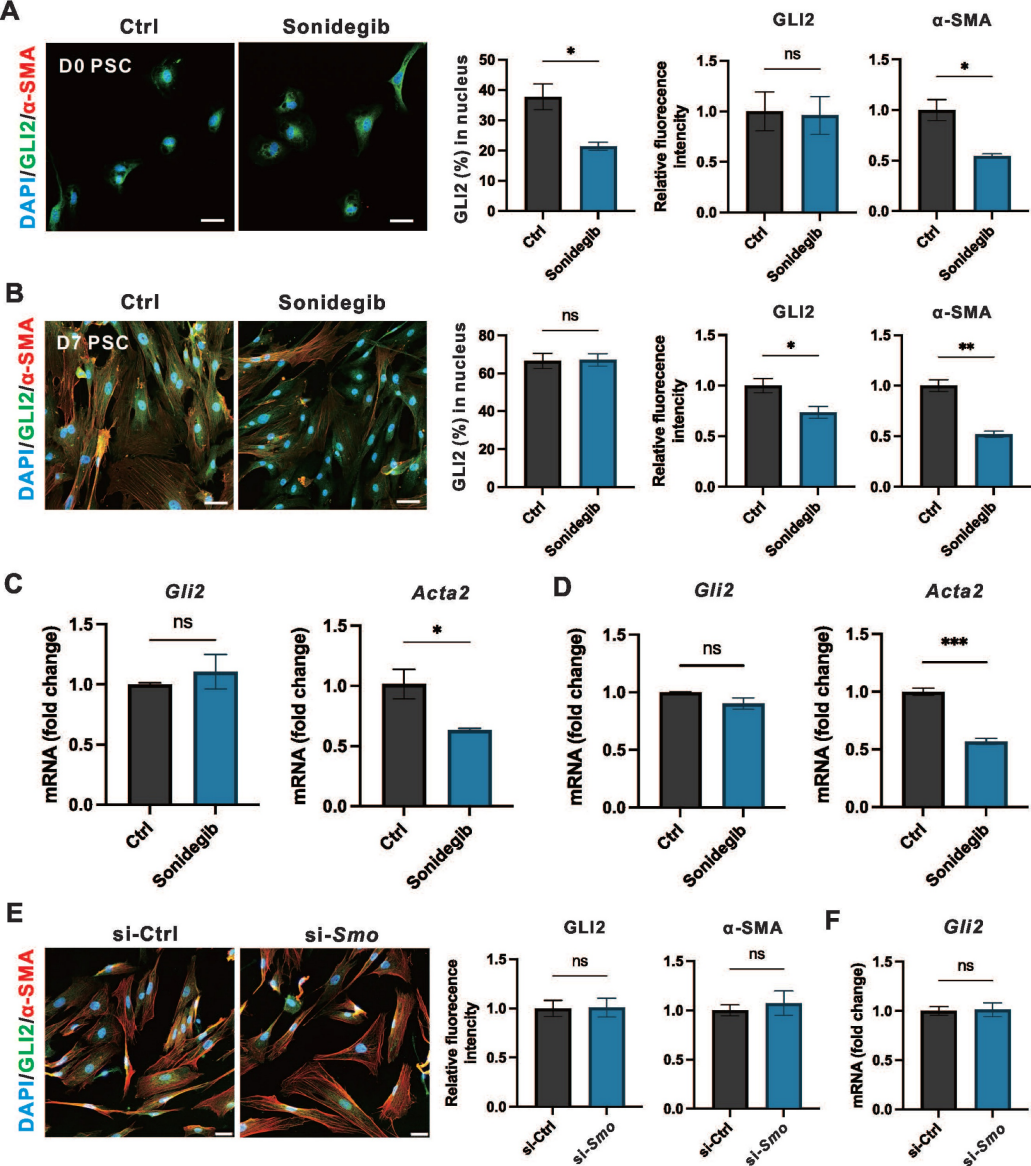
** SMO inhibition is insufficient to affect the expression of GLI2.** (A, B) Representative immunofluorescence images showing α-SMA (red) and GLI2 (green) in primary PSCs treated with Sonidegib, a SMO inhibitor, for 12 hours (A) and 4 days (B) (n = 4). Nuclei were stained with DAPI (blue). Scale bar: 20 µm. The percentage of GLI2 in the nuclear area was quantified (n = 4). (C, D) mRNA expression levels of *Gli2* and *Acta2* in PSCs treated with Sonidegib for 12 hours (C) and 4 days (D) (n = 3). (E) Representative immunofluorescence images showing α-SMA (red) and GLI2 (green) in PSCs following *Smo* silencing. Nuclei were stained with DAPI (blue). Scale bar: 20 µm. GLI2 and α-SMA fluorescence intensities were quantified (n = 3). (F) mRNA expression levels of *Gli2* in PSCs following *Smo* silencing. All data are presented as mean ± SEM. Statistical significance: ^∗^p < 0.05, ^∗∗^p < 0.01; ns, not significant.

We observed that PSC-specific deletion of *Smo* was not sufficient to alleviate fibrosis and the severity CP. To investigate whether SMO influences GLI2 activation or expression during PSC activation, we evaluated the effects of SMO inhibition as it functions as an upstream regulator of GLI2.^34^ To explore the short-term and long-term effects of SMO inhibition on PSC activation, PSCs at day 0 and day 3 were treated with Sonidegib, a SMO inhibitor, for 12 hours and 4 days representing short-term and long-term treatment regimens, respectively. Both short-term (**Figure 5A**) and long-term (**Figure 5B**) treatments reduced PSC activation, with a significant decline in α-SMA expression. However, only short-term Sonidegib treatment of PSCs at day 0 reduced GLI2 nuclear translocation, while SMO inhibition had minimal impact on GLI2 protein expression. Consistently, the *Gli2* mRNA level remained unchanged after both short-term (**Figure 5C**) or long-term (**Figure 5D**) Sonidegib treatments. Additionally, we silenced *Smo* expression in PSCs at day 7 using lentivirus encoding siRNA targeting *Smo* (si-*Smo*). This approach was similarly insufficient to reduce the expression levels of GLI2 protein (**Figure 5E**) and mRNA (**Figure 5F**). Thus, although short-term pharmacological inhibition of SMO reduced GLI2 nuclear translocation, it failed to sustain this effect during long-term treatment. Furthermore, *Smo* knockdown consistently showed no effect on GLI2 expression.

### TGF-β1/SMAD3 signaling promotes the nuclear translocation and expression of GLI2

The results above suggest that SMO activity alone cannot account for the activation and elevated expression of GLI2 observed during PSC activation. Therefore, we examined the role of the TGF-β1/SMAD3 signaling pathway—a key regulator of fibrosis and a known upstream activator of GLI2 via non-canonical Hh signaling—in modulating GLI2 induction and functional activity in PSCs.^35^-^38^

**Figure 6:**
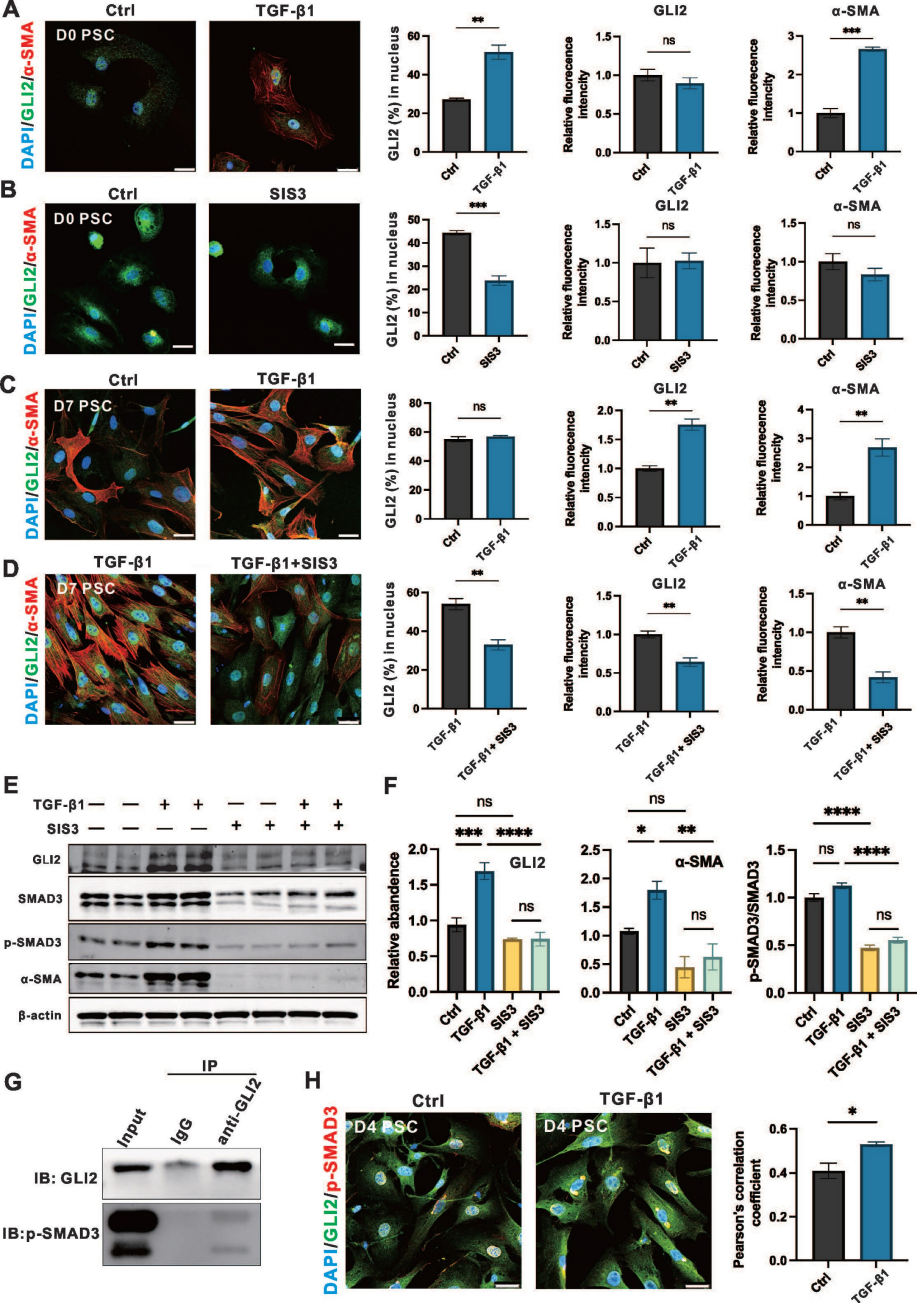
** TGF-β1/SMAD3 promotes the nuclear translocation and expression of GLI2.** (A, B) Representative immunofluorescence images showing α-SMA (red) and GLI2 (green) in primary PSCs treated with TGF-β1 (A) or SIS3 (B) for 12 hours (n = 4). Nuclei were stained with DAPI (blue). Scale bar: 25 µm. The percentage of GLI2 in the nuclear area, as well as GLI2 and a-SMA fluorescence intensities, were quantified (n = 4). (C, D) Representative immunofluorescence images showing α-SMA (red) and GLI2 (green) in primary PSCs treated with TGF-β1 (C) or a combination of TGF-β1 and SIS3 (D) for 4 days (n = 4). Nuclei were stained with DAPI (blue). Scale bar: 25 µm. The percentage of GLI2 in the nuclear area, as well as GLI2 and α-SMA fluorescence intensities were quantified. (E, F) Protein levels of GLI2, SMAD3, p-SMAD3, and α-SMA were assessed by Western blot in primary PSCs treated with single or combined treatments of TGF-β1 and SIS3 for 4 days. β-actin was used to normalize protein levels. GLI2, α-SMA and the p-SMAD3/SMAD3 ratio were quantified across treatment groups (n = 3). (G) Co-IP of endogenous GLI2 and p-SMAD3. Primary PSCs were treated with 10 ng/mL TGF-β1 for 48 hours. (H) Representative immunofluorescence images showing colocalization of GLI2 and p-SMAD3 in TGF-β1-induced PSCs. D2 PSCs were treated with 10 ng/mL TGF-β1 for 48 hours (n = 4). Scale bar: 25 μm. Pearson's correlation coefficient was quantified (n = 4). Western blot lanes were reconstructed, and original blots are provided in Figure S5. All data are presented as mean ± SEM. Statistical significance: ^∗^p < 0.05, ^∗∗^p < 0.01,^ ∗∗∗^p < 0.001,^ ∗∗∗∗^p < 0.0001; ns, not significant.

To evaluate the role of the TGF-β1/SMAD3 signaling on GLI2 activation and expression, day 0 and day 3 PSCs were treated with 1 ng/mL TGF-β1 and 5 μM SIS3, a specific inhibitor of SMAD3, for 12 hours and 3 days. Short-term treatment of PSCs at day 0 with TGF-β1 significantly induced GLI2 nuclear translocation, accompanied by a marked increase in α-SMA expression, although it had minimal effect on GLI2 protein expression levels at day 0 (**Figure 6A**). SMAD3, a downstream transcription factor of TGF-β1, is activated via phosphorylation, and SIS3 inhibits this process by targeting SMAD3 phosphorylation to p-SMAD3.^35^ In day 0 PSCs, SIS3 suppressed GLI2 nuclear translocation but had limited effects on GLI2 and α-SMA expression (**Figure 6B**). While long-term TGF-β1 treatment only minimally affected GLI2 distribution between the cytoplasm and nucleus, it significantly increased the expression of both GLI2 and α-SMA (**Figure 6C**). In contrast, SIS3 reduced GLI2 nuclear translocation and decreased the TGF-β1-induced overexpression of GLI2 and α-SMA (**Figure 6D**). These findings were further supported by Western blot analysis, which showed a significant increase in GLI2 expression following TGF-β1 treatment, an effect reversed by SIS3 (**Figure 6E, 6F and Figure S5A**).

Previous studies have shown that p-SMAD3 can bind GLI2 and promote its nuclear translocation and transcriptional activity. To further explore how TGF-β1/SMAD3 regulates GLI2 activation in PSCs, we performed Co-IP and immunofluorescence assays. Co-IP confirmed a direct interaction between endogenous GLI2 and p-SMAD3 in PSCs following TGF-β1 stimulation (**Figure 6G**). Consistently, immunofluorescence analysis revealed enhanced colocalization of GLI2 and p-SMAD3 upon TGF-β1 treatment (**Figure 6H**).

In addition, we examined whether TGF-β1 influences the expression of other components of the Hh pathway in PSCs. TGF-β1 treatment increased GLI1 mRNA levels and decreased PTCH2 mRNA but did not alter SMO or PTCH1 transcript levels (**Figure S5B**). At the protein level, however, TGF-β1 had no significant effect on the expression of GLI1, SMO, or PTCH2 (**Figure S5C**).

Taken together, these data indicate that GLI2 is activated by the TGF-β1/SMAD3 pathway through both increased expression and nuclear translocation—mechanistically mediated by direct binding of p-SMAD3. These findings support a model in which TGF-β1/SMAD3 promotes Hh pathway activation via a non-canonical route during PSC activation, thereby enhancing fibrogenic responses.

### Gene expression and pathways regulated by GLI2 in PSCs

**Figure 7:**
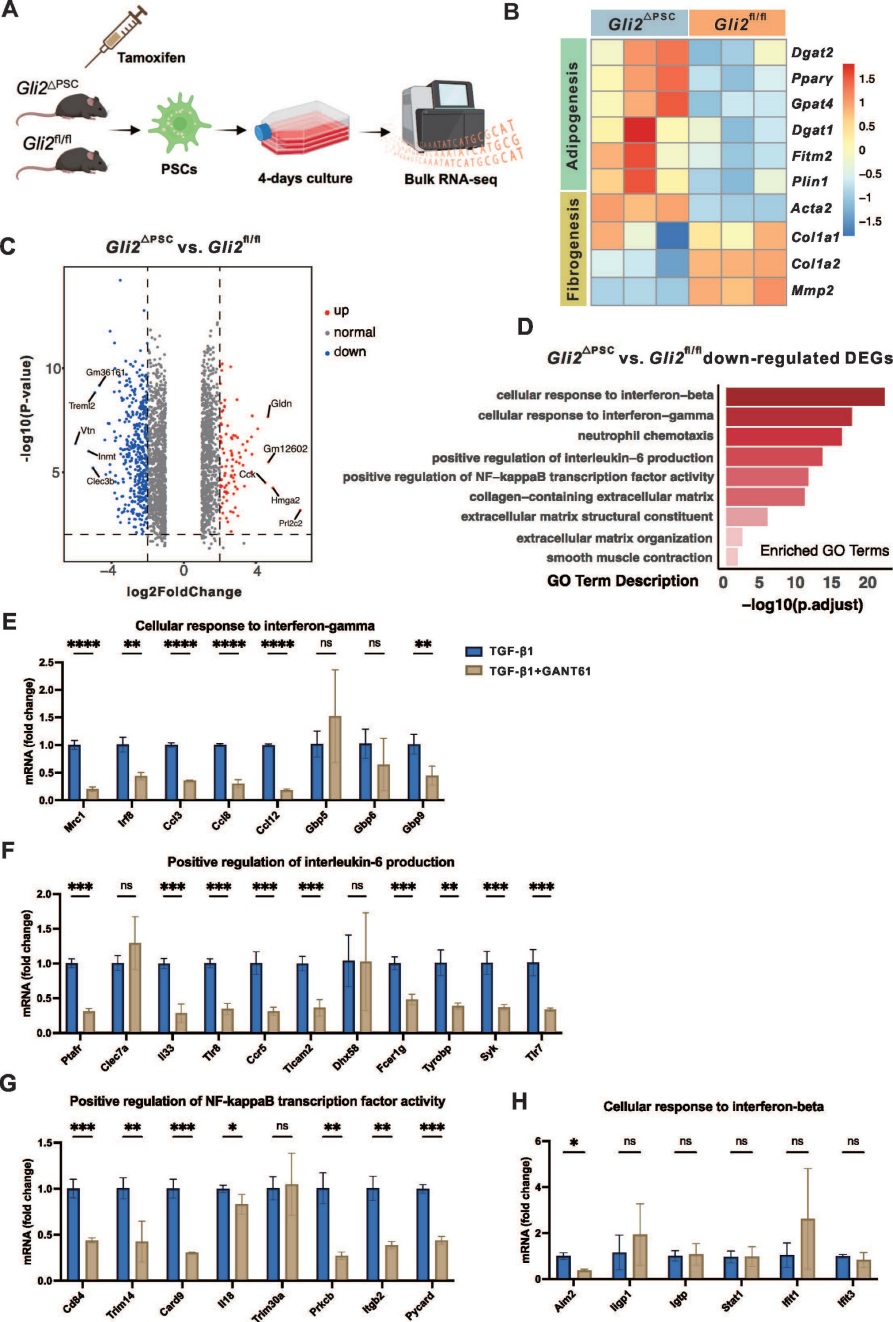
** Gene expression and pathways regulated by GLI2 in PSC.** (A) Schematic representation of the study design for bulk RNA sequencing of PSCs derived from *Gli2*^△PSC^ and *Gli2*^fl/fl^ mice. (B) Heatmap displaying the expression levels of selected adipogenic and fibrotic genes in PSCs derived from *Gli2*^△PSC^ and *Gli2*^fl/fl^ mice. (C) Volcano plots visualizing the differentially expressed genes (DEGs) in PSCs derived from* Gli2*^△PSC^ and *Gli2*^fl/fl^ mice. (D) GO enrichment analysis identifying GLI2-upregulated biological processes and pathways. (E-H) mRNA levels of genes enriched for GO biological processes 'Cellular responses to IFN-γ' (E), 'Positive regulation of IL-6 production' (F), 'Positive regulation of NF-κB transcription factor activity' (G) and 'Cellular response to IFN-β' (H). D2 PSCs were treated with 1 ng/mL TGF-β1 and 5 μM GANT61 for 48 hours (n = 3). All data are represented as mean ± SEM. Statistical significance: ^∗^p < 0.05,^ ∗∗^p < 0.01,^ ∗∗∗^p < 0.001, ns, not significant.

To elucidate the molecular mechanisms underlying the antifibrotic effects of GLI2, we performed bulk RNA sequencing (RNA-seq) of primary PSCs isolated from *Gli2*^△PSC^ and *Gli2*^fl/fl^ mice. The genetically modified mice received tamoxifen injections prior to PSC isolation, and the cells were cultured in vitro for 4 days, a time point when GLI2 expression peaks (**Figure 7A**). Deletion of *Gli2* preserved the lipid metabolic capacity of PSCs, as evidenced by high expression of adipogenic transcription factors such as *Pparγ* and *Dgat2*. Conversely, fibrotic genes, including *Col1a1* and *Col1a2*, were downregulated in PSCs derived from *Gli2*^△PSC^ mice (**Figure 7B**). These changes in adipogenic and fibrotic gene expression suggest that *Gli2* deletion helps maintain PSCs in a quiescent, lipogenic state, preventing their activation.

RNA-seq analysis identified 1,380 downregulated and 860 upregulated differentially expressed genes (DEGs) in PSCs derived from *Gli2*^△PSC^ mice compared to *Gli2*^fl/fl^ mice (**Figure 7C**). The downregulated DEGs were further subjected to functional assessment through gene enrichment analysis for GO biological processes. This analysis highlighted enrichment in categories related to ECM formation, including 'collagen-containing extracellular matrix,' 'extracellular matrix structural constituent,' and 'extracellular matrix organization,' as well as processes like 'smooth muscle contraction,' (**Figure 7D**). Additionally, GO analysis indicated that GLI2 promotes signaling cascades such as interferon-beta (IFN-β), interferon-gamma (IFN-γ), interleukin-6 (IL-6), and nuclear factor-κB (NF-κB) (**Figure 7D**).

To validate these findings, we further examined the expression of key pathway-related genes in wild-type PSCs treated with TGF-β1 and the GLI inhibitor GANT61. In activated PSCs, GANT61 treatment significantly downregulated genes associated with the IFN-γ, IL-6, and NF-κB pathways under TGF-β1 stimulation (**Figure 7E-G**), while expression of IFN-β-related genes remained unchanged (**Figure 7H**). These pharmacological validation experiments in wild-type PSCs were consistent with the transcriptomic data from *Gli2*-deficient PSCs.

Collectively, these findings demonstrate that GLI2 deletion effectively suppresses PSC activation, likely through modulation of ECM-associated processes and crosstalk with multiple pro-fibrotic signaling pathways.

## Discussion

In this study, we investigated the role of TGF-β1/SMAD3-mediated non-canonical Hh signaling in PSC activation and fibrosis during CP progression. Among Hh signaling components, we identified GLI2 as a key effector in the pancreas of CP patients, predominately expressed in PSCs. We found that GLI2 expression was significantly upregulated in aPSCs in both human and experimental CP, correlating with degree of PSC activation. Notably, PSC-specific deletion of GLI2, but not SMO, reduced fibrosis and the severity of CP, demonstrating that GLI2 activation can occur independently of canonical SMO-dependent Hh signaling. Experiments on culture-activated primary PSCs revealed early nuclear translocation and a rapid increase in GLI2 expression during activation, underscoring its central role. Interestingly, TGF-β1/SMAD3 signaling, a predominant pathway in fibrotic diseases including CP, was identified as a driver of GLI2 activation and expression. These findings establish Hh signaling activation as a key mechanism in CP development, suggesting that targeting GLI2 in PSCs could be a promising therapeutic strategy.

The Hh signaling pathway plays a crucial role in regulating cell proliferation, differentiation, and tissue patterning during embryonic development.^18^ In recent years, accumulating evidence has suggested that the Hh pathway is also a key contributor to fibrotic diseases and one of the most important signaling pathways leading to tissue and organ fibrosis.^39^-^41^ Our findings expand the current understanding of the profibrotic role of Hh signaling in CP by showing that GLI2, a transcription factor in the Hh pathway, is upregulated in aPSCs in both mouse CP model and human patients, consistent with its reported overexpression in liver and skin fibrosis.^42^-^44^ During the early stages of PSC activation, primary PSCs exhibit increased GLI2 expression and prominent nuclear translocation. Pharmacological inhibition of GLI2 with GANT61 or siRNA-mediated knockdown of GLI2 significantly reduced PSC activation. Furthermore, in cerulein-induced CP model, *Gli2*^ΔPSC^ mice displayed markedly reduced pancreatic fibrosis compared to *Gli2*^fl/fl^ controls, highlighting the functional importance of GLI2 in driving pancreatic fibrogenesis in vivo. Notably, GLI1, another transcription factor in the Hh pathway and a downstream target of GLI2,^18^ has garnered attention as GLI1-positive cells are a major source of activated fibroblasts in multiple organs.^45^-^48^ Our findings suggest that while GLI1 is upregulated during PSC activation, the changes in GLI1 are less pronounced compared to GLI2 expression. This may be due to the fact that the triggers and mechanisms of injury are disease-specific, but activation of the Hh signaling could be a shared mechanism driving fibroblast activation across various conditions.

Although the importance of Hh signaling is now widely recognized across various diseases, and SMO inhibitors have been approved by the US Food and Drug Administration for the treatment of medulloblastoma and basal cell carcinoma,^49^, ^50^ some clinical studies shown that SMO inhibition is not always effective, with GLI expression persisting despite SMO inhibition.^51^, ^52^ These findings suggest the existence of non-canonical GLI activation, independent of the canonical PTCH-SMO signaling pathway. In the present study, we observed that pancreatic fibrosis was not alleviated in *Smo*^ΔPSC^ mice compared to *Smo*^fl/fl^ controls following CP induction, with PSCs from *Smo*^ΔPSC^ mice still showing high GLI2 expression. These data indicate that deletion of *Smo* in PSCs does not impact downstream GLI2 expression. Moreover, simultaneous CP modeling revealed significantly reduced pancreatic fibrosis in *Gli2*^ΔPSC^ mice compared with *Smo*^ΔPSC^ mice, providing the first direct evidence that GLI2-dependent, SMO-independent non-canonical Hh signaling plays a crucial role in PSC activation and fibrosis progression in CP. In vitro studies further revealed that Sonidegib, a SMO inhibitor, effectively inhibited nuclear translocation of GLI2 in PSCs only upon short-term treatment, but had minimal long-term impact on GLI2 nuclear translocation, GLI2 expression, and PSC activation. Taken together, these findings highlight a critical role for GLI2-mediated non-canonical Hh signaling in pancreatic fibrosis and PSC activation.

Previous reports have indicated that non-canonical GLI2 expression and activity may be driven by TGF-β/SMAD signaling, which predominantly promotes fibrotic diseases including CP.^42^, ^43^, ^53^ In line with these findings, our data show increased GLI2 nuclear localization and expression in PSCs after TGF-β1 treatment, which is mediated by the phosphorylation of SMAD3. Recent research has shown that p-SMAD3 co-localizes with GLI2 in the nucleus, with interactions between GLI2 and p-SMAD3 synergistically enhancing the transcription of downstream target genes.^54^ Our results similarly confirmed that TGF-β1 significantly promoted direct interaction between p-SMAD3 and GLI2. This may explain the TGF-β1-mediated GLI2 nuclear translocation and elevated expression observed in PSC activation. Notably, our findings suggest a phased regulation of GLI2 during PSC activation. In the early stage (within 12 hours of culture), GLI2 nuclear translocation occurs without exogenous TGF-β1 stimulation and is sensitive to the SMO inhibitor Sonidegib, indicating that initial activation is SMO-dependent. However, in the later stage, TGF-β1/SMAD3 signaling becomes the dominant driver of GLI2 nuclear localization and expression, independent of SMO. This temporal shift highlights a transition from canonical to non-canonical Hh pathway regulation, reflecting the dynamic and stage-specific control of GLI2 activity in PSCs. Collectively, these findings suggest that therapeutic strategies aimed at GLI2 may offer greater efficacy than those targeting SMO in blocking Hh pathway activity in PSCs during CP.

Our results indicate the early involvement of GLI2 in PSC phenotypic transition and suggest potential pathways regulated by GLI2. Previous studies have described robust metabolic reprogramming during hepatic stellate cell (HSC) activation, occurring within the first 48 hours of in vitro culture.^55^, ^56^ This phenotypic change in HSCs relies on the induction of aerobic glycolysis, accompanied by suppressed lipogenesis. Inhibiting Hh signaling can prevent this dramatic metabolic reprogramming in HSCs and block their activation.^55^, ^57^ Similarly, our findings in PSCs demonstrate that GLI2 nuclear translocation and increased expression occur within the first 48 hours of in vitro culture, indicating the early involvement of GLI2 in PSC activation. Deletion of *Gli2* in PSCs results in increased lipid synthesis and decreased ECM formation, suggesting that targeting Hh signaling can inhibit metabolic reprogramming and thereby protect against PSC activation. Our data also suggest that key regulators of fibrosis and inflammatory responses, such as IFN-γ, IL-6, and NF-κB may be downstream factors of Hh signaling. Interactions between Hh signaling and these pathways have been observed in various inflammatory diseases and cancers, and require further validation in the context of CP.^58^-^61^

Targeting GLI2 presents a potential therapeutic option for treating CP. However, other several factors must be considered, as Hh signaling is essential for effective tissue regeneration following injury.^62^-^65^ In a mouse model of acute pancreatitis, rapid activation of Hh signaling components was observed following cerulein treatment, with their expression returning to baseline levels after regeneration.^66^, ^67^ Inhibition of Hh signaling, whether through chemical or genetic approaches, resulted in impaired regeneration, characterized by the persistence of metaplastic epithelium expressing markers of pancreatic progenitor cells, indicating an inhibition of redifferentiation into mature exocrine cells.^68^ Consistent with this, our findings demonstrate markedly increased expression of GLI2 and SMO in the pancreatic parenchyma of both CP patients and mouse model of CP. Therefore, therapeutic targeting of Hh signaling requires careful selection of the appropriate treatment window—ideally after parenchymal cell repair completion but before resolution of mesenchymal cell activation.

Interestingly, a recent study using the SMO inhibitor Vismodegib during or after cerulein-induced CP reported significant attenuation of pancreatic fibrosis and promotion of pancreatic regeneration, seemingly contradicting our results where PSC-specific *Smo* deletion failed to alleviate fibrosis.^69^ However, this discrepancy might stem from differences in experimental strategies and timing of intervention. Specifically, global pharmacological inhibition using Vismodegib affects multiple pancreatic cell populations beyond PSCs, possibly eliciting a broader tissue-level response. In contrast, our study's PSC-specific genetic deletion isolates Smo's cell-autonomous effects. Additionally, the beneficial effects observed with Vismodegib differ from earlier findings where SMO inhibitors impaired pancreatic repair post-injury.^68^ Collectively, these contrasting outcomes highlight the complexity of Hh signaling in CP and underscore the importance of carefully choosing therapeutic approaches and intervention timings. Another promising alternative involves targeted delivery systems for pharmaceutical or nucleic acid-based therapeutics directly to PSCs, as demonstrated by recent studies.^70^, ^71^

However, this study has several limitations. First, in the human snRNA-seq dataset, the number of acinar cells from CP patients was extremely low (CP samples, n = 53; healthy controls, n = 79,328). This scarcity resulted in very low detected expression levels of GLI2 transcripts in pancreatic acinar cells, contradicting our immunofluorescence results, which clearly showed elevated GLI2 protein expression in pancreatic parenchymal cells from CP patients. This dramatic cell loss, combined with the RNase-rich environment of acinar cells likely contributed collectively to the apparent underrepresentation of GLI2 mRNA. A second limitation of this study is that we did not evaluate the therapeutic efficacy of pharmacological modulation of the Hh pathway in CP at the in vivo level. Considering the crucial role of Hh signaling in repairing pancreatic parenchymal cell injury, we specifically employed a PSC-specific genetic knockout strategy to isolate and investigate the function of Hh signaling within PSCs.

In summary, the results presented here implicate non-canonical activation of Hh signaling through GLI2 in the progression of pancreatic fibrosis in both CP patients and an animal model. The induction of GLI2 is mediated by TGF-β1/SMAD3 signaling, and its inhibition in PSCs effectively reduces fibrosis both in vivo and in cell culture models. This study provides novel insights into profibrotic role of Hh signaling in PSC activation and identifies GLI2 as a promising therapeutic target for the treatment of CP.

## Supplementary Material

Supplementary figures and table.

## Abbreviations

ACTA2: smooth muscle alpha-actin 2
aPSC: activated pancreatic stellate cell
DEGs: differentially expressed genes
Co-IP: co-immunoprecipitation
COL1A1: collagen type 1 alpha 1
CP: chronic pancreatitis
DMEM: Dulbecco's modified Eagle medium
ECM: extracellular matrix
FBS: fetal bovine serum
FDA: US Food and Drug Administration
GO: Gene Ontology
H&E: hematoxylin and eosin staining
Hh: Hedgehog
HSC: hepatic stellate cell
IFN-β: interferon-beta
IFN-γ: interferon-gamma
IL-6: interleukin-6
NF-κB: nuclear factor-κB
PCR: polymerase chain reaction
PDAC: pancreatic ductal adenocarcinoma
PSC: pancreatic stellate cell
TGF-β/SMAD: transforming growth factor β / small worm and mothers against decapentaplegic
PPARγ: peroxisome proliferator-activated receptor gamma
PTCH: Patched
PDGFRB: platelet-derived growth factor receptor beta
qPSC: quiescent pancreatic stellate cell
SHH: Sonic Hedgehog
SEM: standard error of the mean
SMO: Smoothened
snRNA-seq: single-nucleus RNA sequencing
RT-qPCR: quantitative real-time PCR
RNA-seq: RNA sequencing
