# Supplementary material for: TGF-β1/SMAD3-mediated Non-canonical Hedgehog Signaling Promotes Pancreatic Stellate Cell Activation and Fibrosis in Chronic Pancreatitis
Source: Int J Biol Sci. 2025 Oct 27;21(15):6978–96. doi: 10.7150/ijbs.108149 (PMC12631176; doi:10.7150/ijbs.108149)
Supplement: Supplementary file 1 — Supplementary figures and table. [file ijbsv21p6978s1.pdf]

Supplemental information

TGF-β1/SMAD3-mediated Non-canonical Hedgehog Signaling Promotes Pancreatic Stellate Cell Activation and Fibrosis in Chronic Pancreatitis

Linrui Peng[1†] , Yuchen Hu[1†], Xiaoying Zhang[2†], Chunlu Tan[3], Chan Yang[4], Tingting Liu[2], Pawel E Ferdek[5], Shufen Yin[1], Liu Wang[1], Wei Huang[2,6*], Yuwei Zhang[1*]

1. Department of Endocrinology and Metabolism, Center for Diabetes and Metabolism Research, West China Hospital, Sichuan University, Chengdu, China.
2. West China Centre of Excellence for Pancreatitis, Institute of Integrated Traditional Chinese and Western Medicine, West China-Liverpool Biomedical Research Centre, West China Hospital, Sichuan University, Chengdu, China.
3. Division of Pancreatic Surgery, Department of General Surgery, West China Hospital, Sichuan University, Chengdu, China.
4. Division of Endocrinology and Metabolism, State Key Laboratory of Biotherapy, West China Hospital, Sichuan University and Collaborative Innovation Center of Biotherapy, Chengdu, China.
5. Department of Cell Biology, Faculty of Biochemistry, Biophysics and Biotechnology, Jagiellonian University, Krakow, Poland.
6. West China Biobank, West China Hospital, Sichuan University, Chengdu, China.

† Equal contributors and co-first authors.

* Corresponding authors: Yuwei Zhang, Department of Endocrinology and Metabolism, Center for Diabetes and Metabolism Research, West China Hospital, Sichuan University, Chengdu, China. E-mail: doczhangyw@scu.edu.cn. Wei Huang, West China Centre of Excellence for Pancreatitis, Institute of Integrated Traditional Chinese and Western Medicine, West China-Liverpool Biomedical Research Centre, West China Hospital, Sichuan University, Chengdu, China. E-mail: dr_wei_huang@scu.edu.cn.

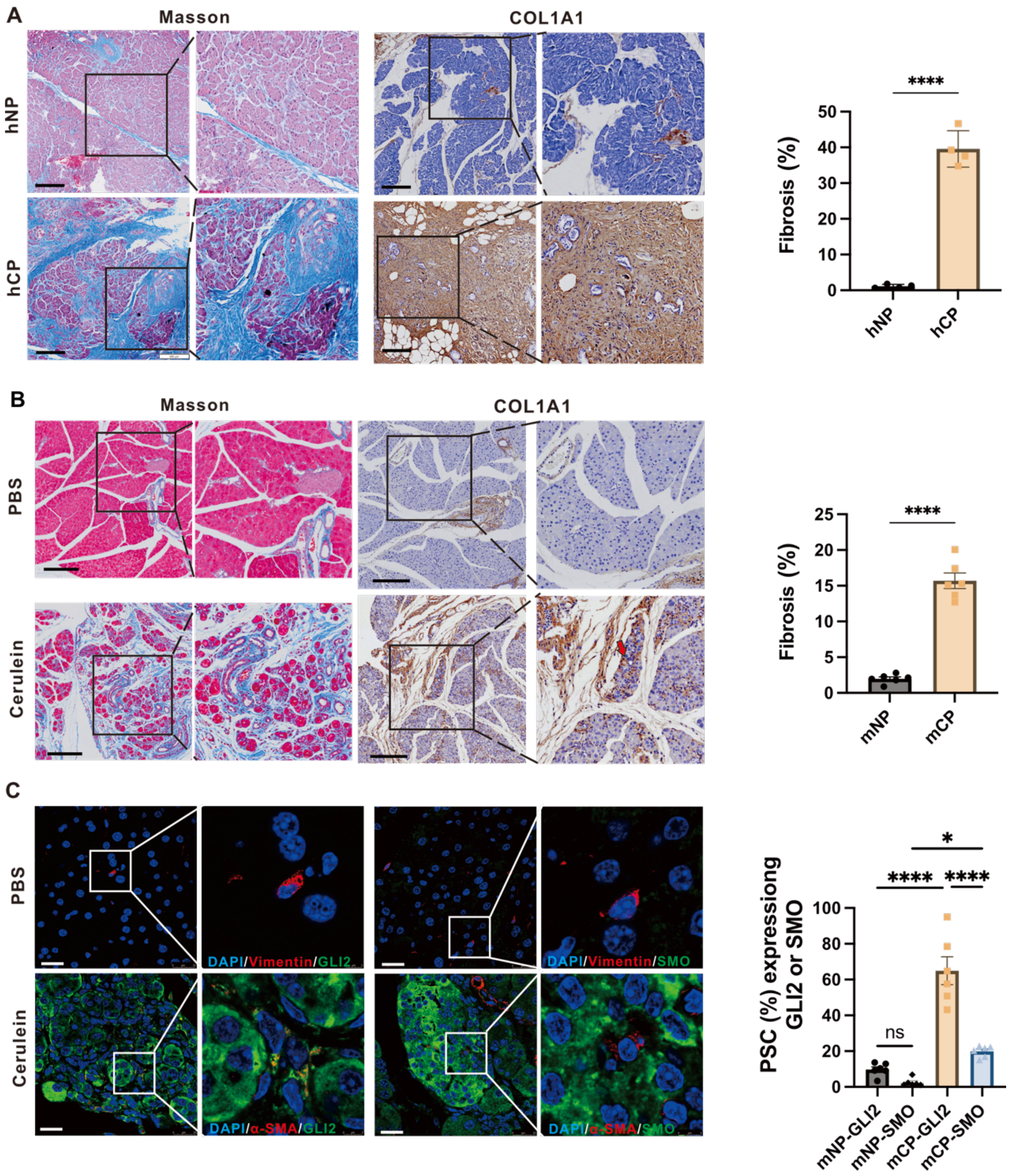

Figure. S1. GLI2 in PSCs is a key component of Hh signaling in CP. (A) Representative images of pancreatic sections stained with Masson's trichrome and Co1la1 in human normal pancreas tissue (hNP, n = 4) and CP pancreas tissue (hCP, n=4). Scale bar, 100 μm. The fibrotic areas were quantified in Masson staining. (B) Representative images of pancreatic sections stained with Masson's trichrome and COL1A1 in mouse normal pancreatic tissue (mNP, n = 6) and CP pancreatic tissue (mCP, n = 6). Scale bar, 100 μm. The fibrotic areas were quantified in Masson staining. (C) Representative dual immunofluorescent images for GLI2 (green) or SMO (green) in mouse normal pancreatic tissue (n = 6) and CP pancreatic tissue (n = 6). Nuclei were stained with DAPI (blue). Scale bar, 25 μm. All data are represented as mean ± SEM. *$p < 0.05$, ****$p < 0.0001$. ns, not significant.

A

B

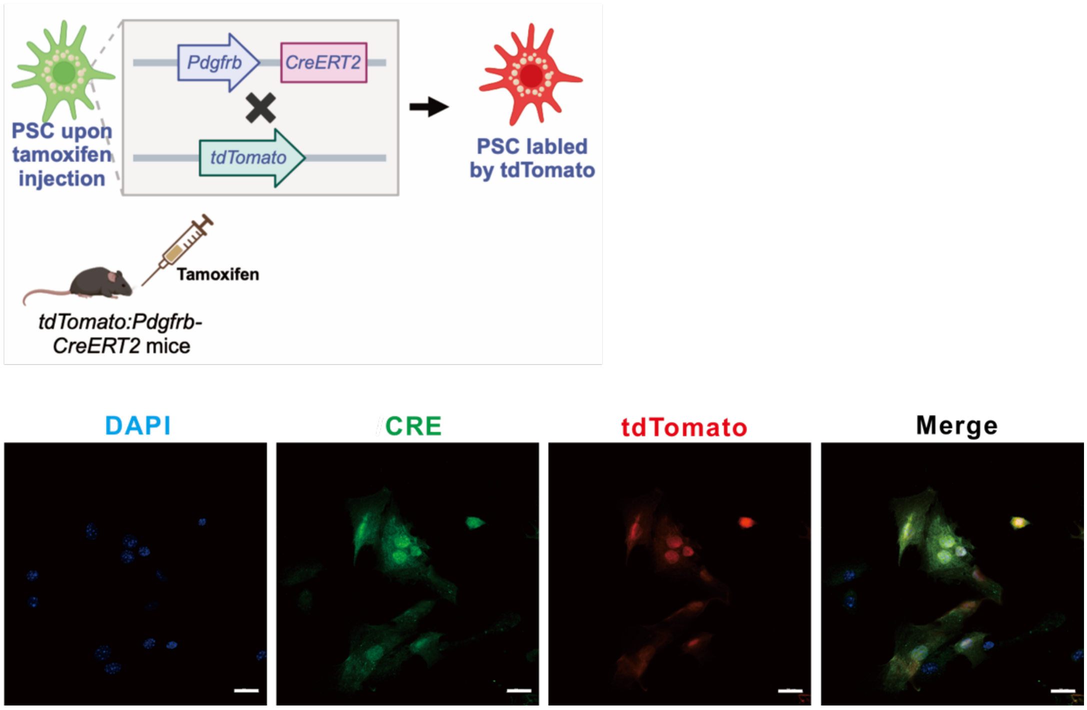

Figure. S2. PSC-specific deletion of *Gli2* alleviates pancreatic fibrosis. (A) Scheme for PSCs labeling with tdTomato. (B) Representative dual immunofluorescent images for CRE (green) and tdTomato (red) in *tdTomato:Pdgfrb-CreERT2* mice derived PSCs. Scale bar, 20 μm.

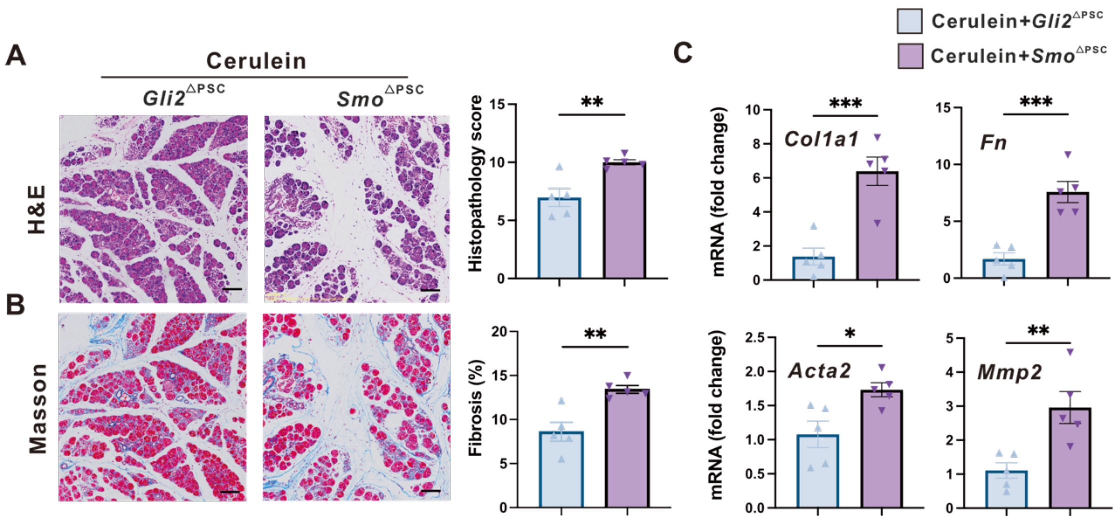

Figure. S3. PSC-specific deletion of *Smo* has minimal impact on fibrosis and severity of CP due to unaffected GLI2 expression. (A-B) Representative images of pancreatic sections stained with H&E (A), Masson's trichrome (B) in *Gli2*$^{\Delta PSC}$ and *Smo*$^{\Delta PSC}$ mice treated with PBS or cerulein. Scale bar, 100 μm. Histopathology scores and fibrotic areas were quantified. (C) Fibrotic markers expression levels in *Gli2*$^{\Delta PSC}$ and *Smo*$^{\Delta PSC}$ with PBS or cerulein. All data are represented as mean ± SEM. *$p < 0.05$, **$p < 0.01$, ***$p < 0.001$, ****$p < 0.0001$.

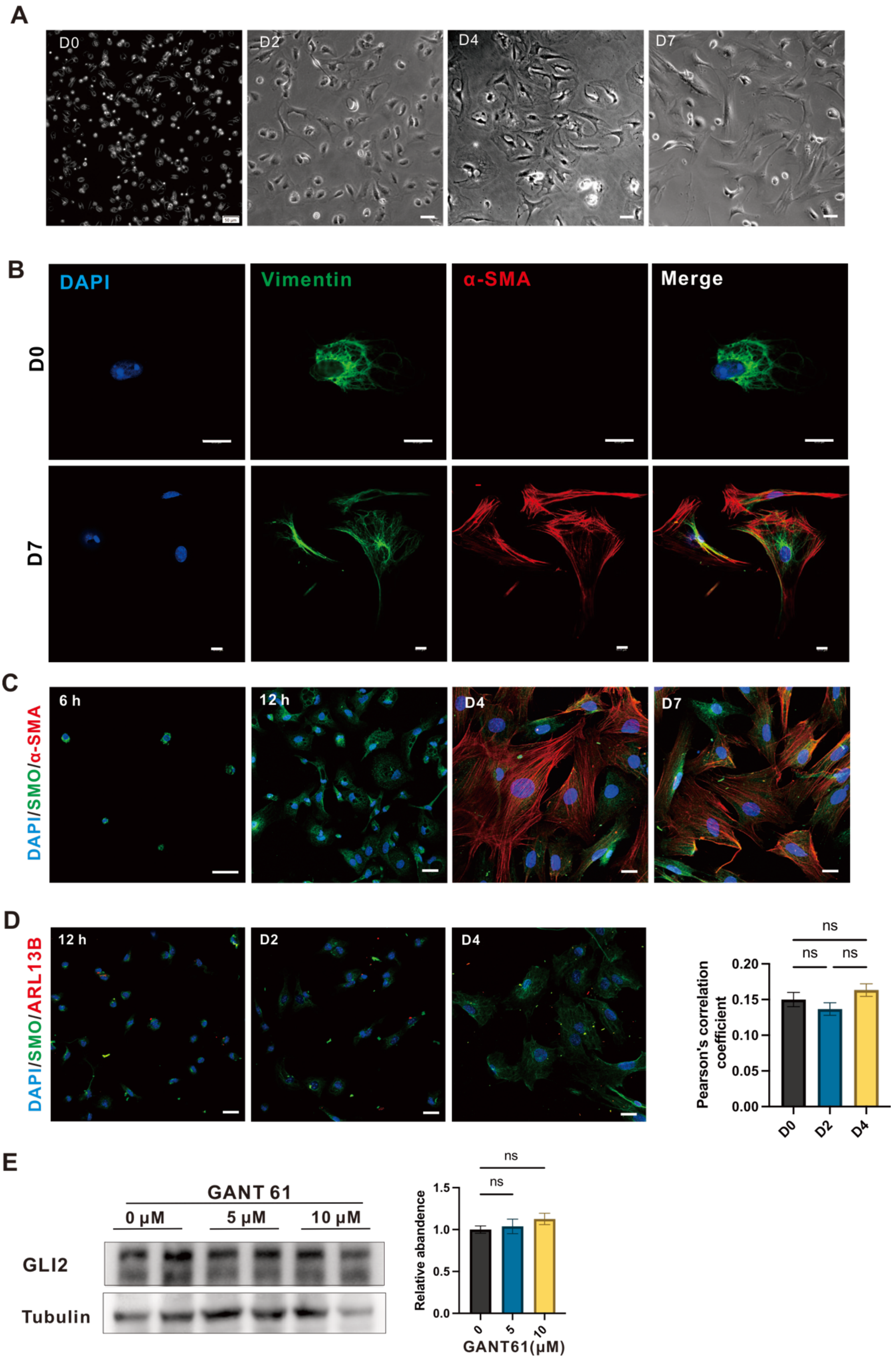

Figure. S4. GLI2 is essential and active in the early stages of PSC activation. (A) Morphological changes in culture-activated PSCs. Scale bar, 50 μm. (B)

Representative dual immunofluorescent images for a-SMA (red) and Vimentin (green) in D0 and D7 PSCs . Nuclei were stained with DAPI (blue). Scale bar, 25 μm. (C) Representative dual immunofluorescent images for a-SMA (red) and SMO (green) in primary PSCs over different culture periods. (D) Representative dual immunofluorescent images for ARL13B (red) and SMO (green) in primary PSCs over different culture periods. Scale bar, 20 μm. Nuclei were stained with DAPI (blue). (E) Expression levels of GLI2 in primary PSCs treated with GANT61. All data are represented as mean ± SEM. ns, not significant.

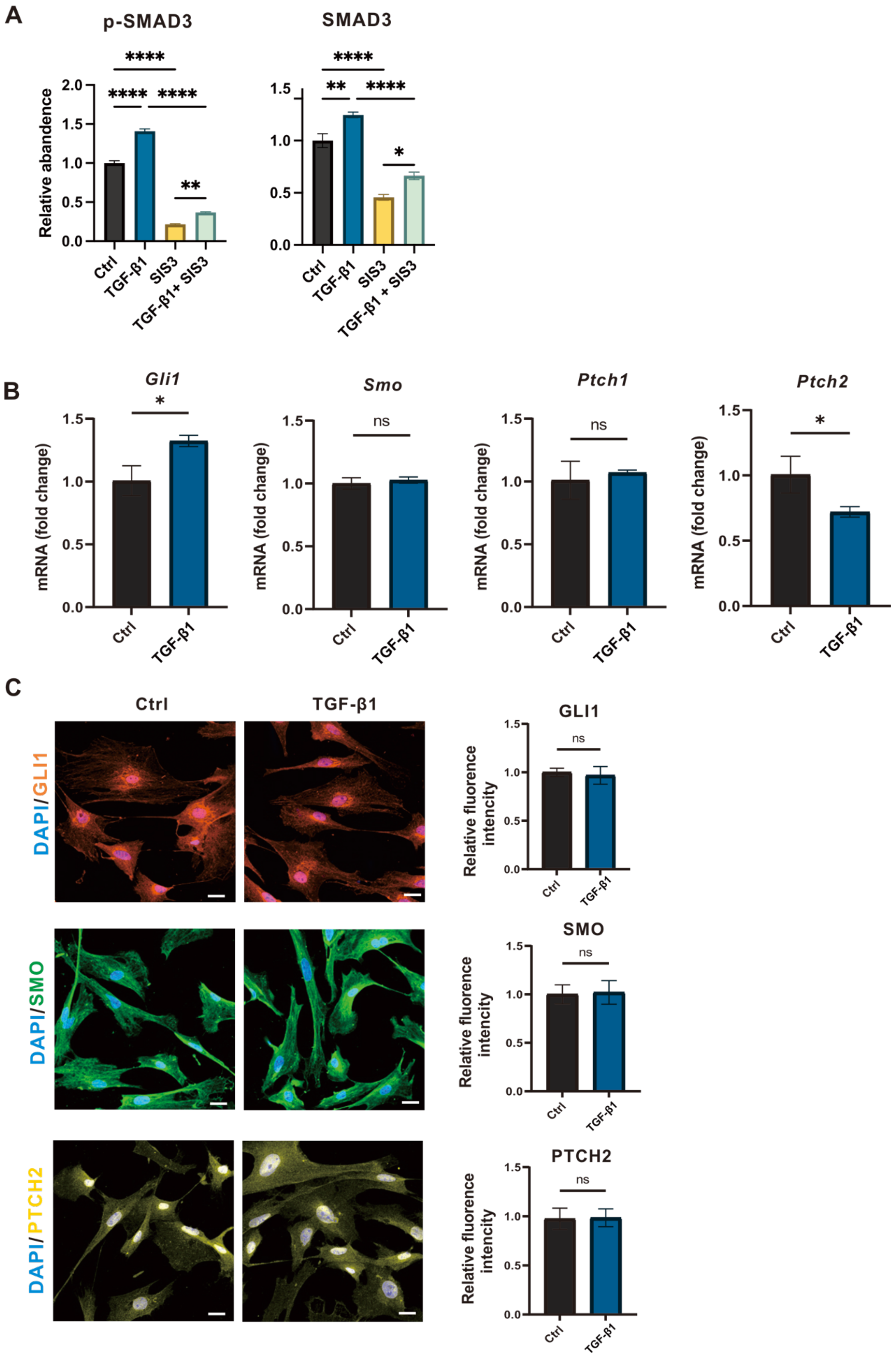

Figure. S5. TGF-β1/SMAD3 promotes the nuclear translocation and expression of GLI2. (A) SMAD3 and p-SMAD3 protein levels were obtained by western blot upon

primary PSC treated with single or combined TGF-β1 and SIS3 treatment for 4 days (n = 4). β-actin was used to normalize protein levels. (B) mRNA levels of *Gli1*, *Smo*, *Ptch1*, and *Ptch2* in PSCs following 24-hour treatment with 10 ng/mL TGF-β1 (n = 3). (C) Representative immunofluorescence images showing expression levels of GLI1 (red), SMO (green), and PTCH2 (yellow) in PSCs treated with 10 ng/mL TGF-β1 for 72 hours. Nuclei were stained with DAPI (blue). Scale bar: 20 μm. Fluorescence intensity of GLI1, PTCH2, and SMO was quantified (n = 4). All data are represented as mean ± SEM. *$p < 0.05$, **$p < 0.01$,***$p < 0.001$,****$p < 0.0001$. ns, not significant.

Western blots for Figure 4

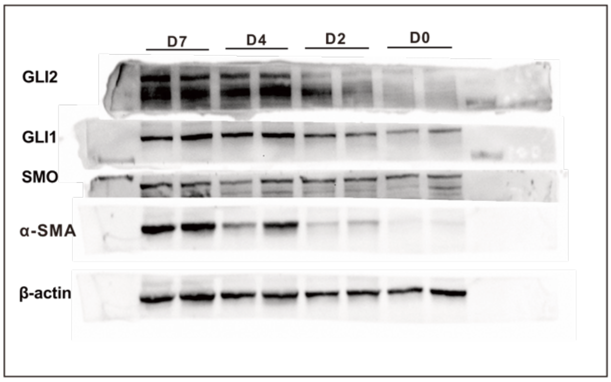

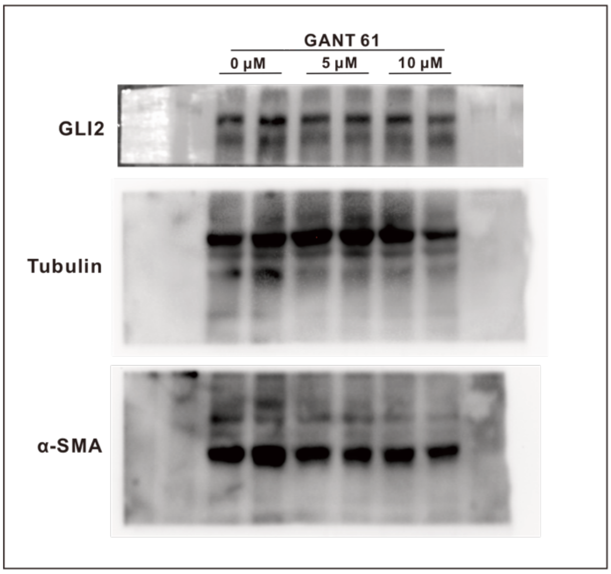

Western blots for Figure 6

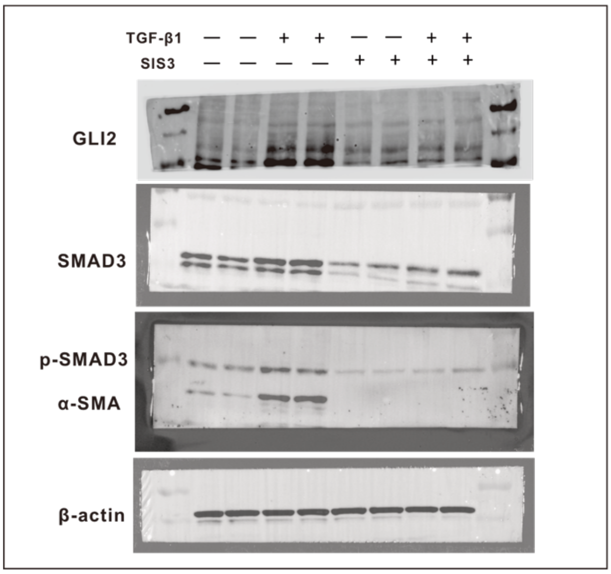

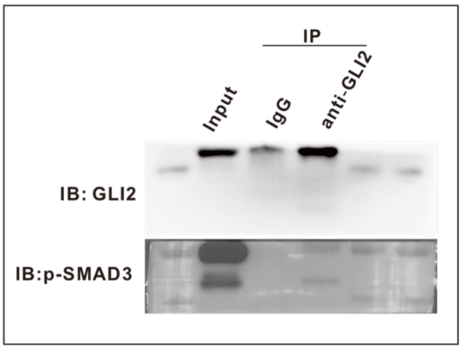

Data. S1. Western blots related to Figure 4 and Figure 6.

Name	5'-3'	Name	5'-3'
Smo_F	GAGCGTAGCTTCCGGGACTA	Ccl12_R	ATCCAGTATGGTCCTGAAGATCA
Smo_R	CTGGGCCGATTCTTGATCTCA	Gbp5_F	CAGACCTATTTGAACGCCAAAGA
Gli2-F	CACCTGCATGCTAGAGGCAAA	Gbp5_R	TGCCTTGATTCTATCAGCCTCT
Gli2-R	AGAAGTCTCCATCTCAGAGGCTCATA	Gbp6_F	GTTCCAGGAAGTAACAAAGGCT
GAPDH_F	TCCACTCATGGCAAATTCAA	Gbp6_R	ATCCCTAGTCTATTCCCAGTGAC
GAPDH_R	TTTGATGTTAGTGGGGTCTCG	Gbp9_F	GGTCACCGGGAATAGACTGG
β-actin_F	GACAGGATGCAGAAGGAGAT	Gbp9_R	GGGCCACACTTGTCATAGCA
β-actin_R	TTGCTGATCCACATCTGCTG	Ptafr_F	TATACTGGGGGTGGTTGCCAA
Acta2_F	CAAGGGATTGGAATTGAGGA	Ptafr_R	GCAGGTCAGCCATAGTGAGATTC
Acta2_R	TGGAAGAAAAATGGGCTTTG	Il18_F	CCTACTTCAGCATCCTCTACTGG
Fn_F	CCTTACACGGTTTCCCATTA	Il18_R	AGGGTTTCTTGAGAAGGGGAC
Fn_R	TTGTCATGGCACCATTTAGA	Il33_F	TCCAACTCCAAGATTTCCCCG
Col1a1_F	TAGGCCATTGTGTATGCAGC	Il33_R	CATGCAGTAGACATGGCAGAA
Col1a1_R	ACATGTTCAGCTTTGTGGACC	Tlr7_F	ATGTGGACACGGAAGAGACAA
Mmp2_F	CAAGTTCCCCGGCGATGTC	Tlr7_R	GGTAAGGGTAAGATTGGTGGTG
Mmp2_R	TTCTGGTCAAGGTCACCTGTC	Tlr8_F	GAAAACATGCCCCCTCAGTCA
Ptch1_F	AAAGAACTGCGGCAAGTTTTTG	Tlr8_R	CGTCACAAGGATAGCTTCTGGAA
Ptch1_R	CTTCTCCTATCTTCTGACGGGT	Ccr5_F	TTTTCAAGGGTCAGTTCCGAC
Ptch2_F	CTCCGCACCTCATATCCTAGC	Ccr5_R	GGAAGACCATCATGTTACCCAC
Ptch2_R	TCCCAGGAAGAGCACTTTGC	Ticam2_F	CGATCAAGACGGCCATGAGTC
Gli1_F	CTCAAACTGCCCAGCTTAACCC	Ticam2_R	CTCGTCGGTGTCATCTTCTGC
Gli1_R	TGCGGCTGACTGTGTAAGCAGA	Dhx58_F	GGAAGTGATCTTACCTGCTCTGG
Iigp1_F	CAGGACATCCGCCTTAACTGT	Dhx58_R	TTGCCTCTGTCTACCGTCTCT
Iigp1_R	AGGAAGTAAGTACCCATTAGCCA	Clec7a_F	GACTTCAGCACTCAAGACATCC
Aim2_F	GTCACCAGTTCCTCAGTTGTG	Clec7a_R	TTGTGTCGCCAAAATGCTAGG
Aim2_R	CACCTCCATTGTCCCTGTTTTAT	Fcer1g_F	ATCTCAGCCGTGATCTTGTTCT
Igtp_F	CTCATCAGCCCGTGGTCTAAA	Fcer1g_R	ACCATACAAAAACAGGACAGCAT
Igtp_R	CACCGCCTTACCAATATCTTCAA	Tyrobp_F	GAGTGACACTTTCCCAAGATGC
Stat1_F	TCACAGTGGTTCGAGCTTCAG	Tyrobp_R	CCTTGACCTCGGGAGACCA
Stat1_R	GCAAACGAGACATCATAGGCA	Card9_F	CTCTGTGCAGGAGGGTAAGC
Ifit1_F	CTGAGATGTCACTTCACATGGAA	Card9_R	TCCGTAGGGAGAAGATGGTG
Ifit1_R	GTGCATCCCCAATGGGTTCT	Syk_F	CTACCTGCTACGCCAGAGC
Ifit3_F	CCTGTGTACCACAAGGGAACT	Syk_R	GCCATTAAGTTCCCTCTCGATG
Ifit3_R	CTGGGGCCACACGAAAGAAA	Pycard_F	CTTGTCAGGGGATGAACTCAAAA
Mrc1_F	CTCTGTTCAGCTATTGGACGC	Pycard_R	GCCATACGACTCCAGATAGTAGC
Mrc1_R	CGGAATTTCTGGGATTCAGCTTC	Cd84_F	ATATAGCTGGAGTCCCTTTGGAG
Irf8_F	CGGGGCTGATCTGGGAAAAT	Cd84_R	AAAGAGCACGGCCAATCCTC

Name	5’-3’	Name	5’-3’
Irf8_R	CACAGCGTAACCTCGTCTTC	Trim14_F	GTGCGTGTGCAGAAGCTAATC
Ccl3_F	TTCTCTGTACCATGACACTCTGC	Trim14_R	CTGCGTAAACCTTGAGCCTTT
Ccl3_R	CGTGGAATCTTCCGGCTGTAG	Trim30a_F	CTGTGAGTGCTGATTGTAACCA
Ccl8_F	TCTACGCAGTGCTTCTTTGCC	Trim30a_R	ACTCGGCATACAGGGCAGT
Ccl8_R	AAGGGGGATCTTCAGCTTTAGTA	Prkcb_F	GTGTCAAGTCTGCTGCTTTGT
Ccl12_F	ATTTCCACACTTCTATGCCTCCT	Prkcb_R	GTAGGACTGGAGTACGTGTGG
Igtp2_R	CCTGGTCCAGTGAAGTTCAGC	Igtp2_F	CAGGAATGCACCAAGTACAAAGT

Table. S1. Primers used for gene amplification. Related to Figure 2,3,4 and Figure 5.
